# Determining correlates of the average number of cigarette smoking among college students using count regression models

**DOI:** 10.1038/s41598-020-65813-4

**Published:** 2020-06-01

**Authors:** Parami Sharareh, Tapak Leili, Moghimbeigi Abbas, Poorolajal Jalal, Ghaleiha Ali

**Affiliations:** 1 Department of Biostatistics, School of Public Health, Hamadan University of Medical Sciences, Hamadan, Iran; 2 Modeling of Noncommunicable Diseases Research Center, Hamadan University of Medical Sciences, Hamadan, Iran; 3 Department of Biostatistics, School of Public health, Alborz University of Medical Sciences, Alborz, Iran; 4 Department of Epidemiology, School of Public Health, Hamadan University of Medical Sciences, Hamadan, Iran; 5 Research Center for Health Sciences, Hamadan University of Medical Sciences, Hamadan, Iran; 6 Department of Psychiatry, School of Medicine, Hamadan University of Medical Sciences, Hamadan, Iran; 7 Behavioral Disorders and Substance Abuse Research Center, Hamadan University of Medical Sciences, Hamadan, Iran

**Keywords:** Medical research, Risk factors

## Abstract

College students, as a large part of young adults, are a vulnerable group to several risky behaviors including smoking and drug abuse. This study aimed to utilize and to compare count regression models to identify correlates of cigarette smoking among college students. This was a cross-sectional study conducted on students of Hamadan University of Medical Sciences. The Poisson, negative binomial, generalized Poisson, exponentiated-exponential geometric regression models and their zero-inflated counterparts were fitted and compared using the Vuong test (α = 0.05). A number of 1258 students participated in this study. The majority of students were female (60.8%) and their average age was 23 years. Most of the students were non-smokers (84.6%). Negative binomial regression was selected as the most appropriate model for analyzing the data (comparable fit and simpler interpretation). The significant correlates of the number of cigarettes smoked per day included gender (male: incident-rate-ratio (IRR = 9.21), birth order (Forth: IRR = 1.99), experiencing a break-up (IRR = 2.11), extramarital sex (heterosexual (IRR = 2.59), homosexual (IRR = 3.13) vs. none), and drug abuse (IRR = 5.99). Our findings revealed that several high-risk behaviors were associated with the intensity of smoking, suggesting that these behaviors should be considered in smoking cessation intervention programs for college students.

## Introduction

Smoking is considered as one of the major causes of mortality and morbidity worldwide^1^ with an estimated 8 million deaths from smoking tobacco and cigarette annually; where more than 7 million of these deaths are caused directly by tobacco consumption^2^. Smoking is among the major causes of preventable deaths from respiratory and cardiovascular diseases and many other different types of cancers^3^. Smoking is also responsible for endangering mental health in addition to physical health and can underlie opium addiction^4^. Smoking even one cigarette a day can increase one’s heart rate and blood pressure^5^. According to the World Health Organization, there are about 1.1 billion smokers worldwide with 80% living in low- and middle-income countries, where the burden of illness/death related to tobacco is heaviest^2^. It has been reported that the onset age of smoking is diminishing^6,7^. Therefore, smoking has become a focal point of attention.

College students, as a large part of young adults in every country, are a special vulnerable group to embracing several risky behaviors including smoking and drug abuse^8^. In developing countries, a wide range of the prevalence of cigarette smoking has been reported among college students. For example, the estimates of the current tobacco smoking prevalence (daily and occasional smoking in the past 30 days preceding the study) among university students were 60.2% in Bangladesh, 30% in Palestine, 26.7% in India 22.2% in Saudi Arabia, and 20.7% in Syria^9–13^. Among Iranian college students, this quantity varies between 13.4% and 39.9% across different provinces throughout the country^14^. For many, college can be an interesting period of life. However, it can also be the onset of risky behaviors for college students due to being exposed to substantial pressures, including financial and academic ones such as long hours of study, living away from home for the first time, and irregular sleep patterns^15–18^.

Smoking cigarette is a precarious and risky behavior; as the smoker is exposed to over 7,000 chemicals^19^ (carcinogens and other types of toxins identified in cigarette smoke of which 69 are the causes of cancer and at least 250 are harmful to health)^2^. It is reported that “On average, each cigarette smoked cuts someone’s life by 11 minutes and stopping smoking is arguably the single most important change that smokers can make to improve their health”^20,21^. Therefore, it is evident that smoking greater numbers of cigarettes may be associated with more serious consequences. It has been reported that the risk of dying from respiratory and heart diseases is, 3 fold and 2 fold respectively, higher for smokers in comparison with non-smokers, but it is more pronounced in heavy smokers (5 fold higher for both respiratory and heart diseases)^22,23^. Moreover, the risk of miscellaneous health outcomes, including oral hygiene (e.g. tooth loss) and obesity in heavy smokers, is higher than non-smokers^24–26^. Furthermore, heavier smokers are more dependent on nicotine and are also less likely to be successful during smoking cessation programs. Thus, they may continue smoking into older adulthood compared with lighter smokers^27^. The majority of individuals who start smoking in adolescence/young adulthood tend to develop regular cigarette smoking later in their life^28,29^ and ceasing smoking is more difficult for them, when they have been smoking for a long time^29,30^. Several studies have been conducted to determine related risk factors of smoking among college students^8,31,32^. However, few studies can be found on investigating the correlates of the intensity of smoking among college students, which highlights the importance of considering the number of cigarettes smoked per day as a count response variable and investigating its correlates.

Under the concept of generalized linear models, there are several regression models for analyzing count data. Poisson regression (and its zero-inflated form known as zero-inflated Poisson regression (ZIP)) and negative binomial regression (NB; and its zero-inflated form known as zero-inflated negative binomial regression (ZINB)) are the two first choices for modeling counts. However, the former has an unreal assumption of equal variance and mean of the distribution and the later can be inefficient at capturing overdispersion (greater variance compared with the mean). There are also other choices for analyzing count data, including generalized Poisson (GP) or zero-inflated generalized Poisson (ZIGP) as well as a newly developed regression model known as exponentiated-exponential geometric regression (EEGR) ant its zero-inflated form (ZIEEGR), that have been shown to have a great performance in modeling count data in different fields^33^. A number of studies have been conducted on the tobacco consumption to compare some of these models including Poisson regression, ZIP regression, NB regression, ZINB regression, and NB hurdle (HUNB) regression^34^. Nevertheless, the performance of a model is data dependent and there is a need to investigate and to compare the performances of different models in different datasets.

Since the age of smoking onset has decreased in recent years^7,35^, especially in developing countries like Iran as it has been reported to be between 17.2 and 23.5 years in Iran^36^, it is important to identify smoking correlates among college students more reliably using an appropriate statistical method (that is well-fitted to the data) to help policymakers and governors in educational planning in universities to provide appropriate interventional programs. These programs may help students to avoid smoking or stop tobacco use, reducing the probability of being a smoker later in their lifespan^37^. This study aimed to examine and to compare different existing count regression models to identify potential correlates of the number of cigarettes smoked per day by the students in Western Iran. The results of this study may provide an infrastructure for health care specialist to design interventions to help all smokers to quit.

## Material and methods

### Data

In this cross-sectional study (approved by “The Ethics Committee of the Hamadan University of Medical Sciences”; NO. IR.UMSHA.REC.1398.076), a dataset related to the college students (passed at least one semester) studying at the Hamadan University of Medical Sciences, Hamadan, Iran, was used. All methods were carried out in accordance with relevant guidelines and regulations. The data were collected from January to May 2016 by a proportional random sampling method using a self-administered questionnaire (including demographic characteristics, personal information, and behavioral risk factors) as well as the Persian version of the General Health Questionnaire-28 (GHQ-28)^38^. For a complete description of the data collection process, see this paper^39^.

#### Outcome variable

The number of cigarettes smoked per day by each student was considered as the outcome variable. This study used the response to this question to identify the correlates of smoking intensity among students of Hamadan University of Medical Sciences.

#### Explanatory variables

Other information was used as potential explanatory variables as follows: 1) personal and demographic characteristics (including sex (male/female), age, marital status (never married/married/divorced), city (hometown/surrounding towns/towns of other provinces), residence (dormitory/parents’ house), birth order (first, second, etc.), parental/maternal educational level (high school Diploma, BSc, MSc, PhD); 2) educational information (including college (study field), the average grade of the previous semester and student’s education level (BSc, MSc, PhD)); 3) if the student has an interest in the discipline/study field (Yes/No; this question evaluated whether the student has selected the field of education based on his/her interest or according to the job opportunity.) and being optimistic about the future; 4) behavioral variables (including having a boy/girlfriend, experiencing a break-up (Yes/No), having sexual intercourse (homosexual, heterosexual, none), illicit drug use (opium/psychedelic ever; psychedelic is a substance that alters cognition/perception in a way that often produces some kind of hallucination or change in how the user perceives reality), having suicide thought ever, having a suicide attempt ever, using social media during a day; and 5) a validated Persian version of the GHQ-28 (Cronbach’s alpha = 0.87 for the present study). This questionnaire provides scores ranged from 0 to 84, with a cutoff point of 23 that determines if a student has/has not psychiatric distress, based on the Iranian version of the questionnaire (21). Moreover, the GHQ-28 has four subscales including somatic symptoms (items 1–7); anxiety/insomnia (items 8–14); social dysfunction (items 15–21), and severe depression (items 22–28). All variables were selected based on literature review and previous studies. The description of the selected explanatory variables was presented in Table 1.

**Table 1:** Demographic and personal characteristics of the college students participated in the study.

Variables	Number	Percent	Variables	Number	Percent
Gender			The average grade of the previous semester		
Male	493	39.2	A (19–20)	35	2.8
Female	765	60.8	B (17–19)	439	34.9
Age group (yr)			C (15–17)	614	48.8
18–21	552	43.9	D (<15)	170	13.5
22–25	550	43.7	Interested in the discipline		
26–29	112	8.9	Yes	1030	81.9
+30	44	3.5	No	228	18.1
Birth order			Optimistic about future		
First	445	35.4	Yes	998	79.3
Second	396	31.5	No	260	20.7
Third	228	18.1	Having a boyfriend/girlfriend		
Forth	189	15.0	Yes	650	51.7
Marital status			No	608	48.3
Single/divorced	1094	87.0	Experiencing a break-up		
Married	164	13.0	Yes	419	33.3
Drug abuse (opium/Psychedelic)			No	839	66.7
Yes	123	9.8	Having sexual intercourse		
No	1135	90.2	None	1065	84.7
Residence			Heterosexual	94	7.5
Dormitory	888	70.6	Homosexual	99	7.9
Parents' house	370	29.4	City		
Father's educational level			Hometown (Hamadan)	381	30.3
Diploma	592	47.1	Surrounding towns	396	31.5
BSc	435	34.6	Towns of other provinces	481	38.2
MSc	166	13.2	Suicide thought lifetime		
MD	65	5.2	Yes	166	13.2
Mother's educational level			No	1092	86.8
Diploma	804	63.9	Suicide attempt lifetime		
BSc	313	24.9	Yes	77	6.1
MSc	108	8.6	No	1181	93.9
MD	33	2.6	Using social media		
Student's education level			Yes	1106	87.9
BSc	599	47.6	No	152	12.1
MSc	96	7.6	GHQ28		
MD	519	41.3	Has not psychiatric distress	741	58.9
PhD	44	3.5	Has psychiatric distress	517	41.1

### Statistical models

#### Poisson regression

The Poisson probability distribution is as follows:

1 $$f(y;\lambda )=\frac{{e}^{-\lambda }{\lambda }^{y}}{y!}\,y=0,1,2,3,\mathrm{..}.$$

with $E(Y)=Var(y)=\lambda$, where $\lambda$ stands for the mean (and variance) of the response variable. To investigate the effect of explanatory variables, the canonical link (here logarithm of $\lambda$) is used to relate mean parameter $\lambda$ to the covariates ($\log (\lambda )=x{\prime} \beta$).

#### Negative binomial regression

The probability mass function of the negative binomial distribution is as follows:

2 $$f(y;\lambda ,\alpha )=\frac{\Gamma (y+\frac{1}{\alpha })}{\Gamma (\frac{1}{\alpha })\varGamma (y+1)}{\left(\frac{1}{(1+\alpha \lambda )}\right)}^{\frac{1}{\alpha }}{\left(1-\frac{1}{(1+\alpha \lambda )}\right)}^{y}y=0,1,2,\mathrm{..}.$$

with mean and variance of $E(y)=\lambda$ and $V(y)=\lambda +(\alpha {\lambda }^{2})$, respectively. The canonical link function of the NB regression is $\log (\lambda )=x{\prime} \beta$. The parameter $\alpha$ is called dispersion (over-dispersion) parameter^34^.

#### Generalized poisson regression

The probability function of y with generalized distribution is given as follows:

3 $$f(y;\lambda ,\,\alpha )={\left(\frac{\lambda }{1+\alpha \lambda }\right)}^{y}\frac{{(1+\alpha y)}^{y-1}}{y!}\exp \left[\frac{-\lambda (1+\alpha y)}{1+\alpha \lambda }\right]\,y=0,1,2,\mathrm{..}.$$

with mean and variance of $E(y)=\lambda$ and $Var(y)=\lambda {(1-\alpha \lambda )}^{2}$, respectively. This distribution can handle modeling of under/overdispersed ($\alpha {\mathbb{\in }}{\mathbb{R}}$ is the dispersion or heterogeneity parameter) data. The link function of the GP is $\lambda =\exp (x{\prime} \beta )$.

#### Exponentiated-exponential geometric regression

The exponentiated-exponential distribution is a unimodal and right-skewed distribution. The probability function of Y_i_ with EEG distribution is given as follows:

4 $$f(y;\theta ,c)={(1-{\theta }^{y+1})}^{c}-{(1-{\theta }^{y})}^{c}\,y=0,1,2,\mathrm{..}.$$

where c > 0 (c affects the shape of the distribution and over/under dispersion; so that the values ≤2 are related to the over-dispersion, while the values greater than 2 are related to both over/under/equi-dispersed distributions) and $0 < {p}^{\lambda }=\theta  < 1$. This distribution does not have a mean and variance in closed-forms. Therefore, Famoy *et al*. suggested that the regression problem should be handled through $\theta ({x}_{i})={\theta }_{i}=f({x}_{i},\beta )={e}^{{x{\prime} }_{i}^{{\prime} }\beta }/1+{e}^{{x}_{i}^{{\prime} }\beta }$ function^33^.

#### Zero-Inflated models

Sometimes, the data consist of many zeros that cannot be handled using the above distributions. All distributions of Poisson, NB, GP, and EEGR can be considered as mixture models called zero-inflated (ZI) models to account for the excess zero counts. A ZI model is based on a logistic regression (typically with a logit link) to predict which class the zero belongs to. The general form of a ZI distribution is as follows:

5 $$f({y}_{i}|\lambda )=\{\begin{array}{c}\phi +(1-\phi )f\,({y}_{i}=0){y}_{i}=0\,{\rm{Logit}}\,{\rm{section}}\\ (1-\phi )f({y}_{i})\,{y}_{i}=1,2,\mathrm{..}.\,{\rm{Standard}}\,{\rm{model}}\,{\rm{section}}\end{array}$$

where f(y) stands for the count distribution and the parameter $\phi$ is the uncertainty parameter (mixing proportion).

### Model fitting and selection

The average daily number of cigarettes (count), smoked by the students, was modeled as a function of gender, age and other explanatory variables using Poisson regression, NB regression, generalized Poisson regression, EEG regression and their zero-inflated counterpart regression models. The same explanatory variables were included in both parts (the logit and count components) of the zero-inflated models. In the EEGR model, we assumed that the shape parameter c is a nuisance parameter. We utilized a multivariate approach for model fitting. Therefore, all the variables were considered in all the models. The Vuong test^40^ (based on BIC and AIC) was used to conduct all the pairwise comparisons between different models to see which one provides a better fit to the data. This test produces a z-statistic, where a value >1.96 supports the alternative assumption that the first model fits the data better and a value <−1.96 indicates that the second model provides a better fit to the data. Data were analyzed using PROC NLMIXED in SAS, version 9.4 (SAS Institute, Inc., Cary,NC). The SAS codes for different count regression models and R codes for the Voung test, provided by the authors, are included in the supplementary file.

### Ethics approval and consent to participate

This study was submitted to and approved by the Ethical Committee of Hamadan University of Medical Science (IR.UMSHA.REC.1398.076). All participant signed an informed consent.

## Results

A number of 1258 students participated in this study. About 84% (1064 out of 1258 participants) of the students were nonsmokers and the average daily cigarettes smoked was 4.36 (standard deviation = 5.04). Table 1 shows the characteristics of the students participated in this study. According to the results, shown in Tables 1, 60.8% of the students were female. The average age of the students was 22.54 years (SD = 3.35) with the majority aged 18–21 years (43.9%). Most of the students participated in the study were single/divorced (87%). About 35% of the students were first-born children, the majority of them lived in the dormitory (70.6%), and 29.4% of them were indigenous. Most of the students (88.9%) were BSc/MD students and were interested in their discipline (81.9%). The education level of most of the parents was a high school diploma (63.9% of mothers and 47.1% of fathers), 51.7% of the students had a boy/girlfriend and 33.3% of them experienced a break-up, 7.9% (7.5%) of the students had homosexual intercourse (heterosexual intercourse), 79.3% of them were optimistic about the future and 13.2% (6.1%) of them had suicidal thought (attempt) during their lifetime. 9.8% of the students had a history of drug abuse (ever) and 87.9% used social media, 41.1% of the students had psychiatric distress in terms of GHQ-28. Summary statistics of the GHQ-28 subscales as well as the total score for the college students were also provided in Table 2. As seen, the average and standard deviation of the general health of the students participated in this study was 22.72 and 14.80, respectively.

**Table 2:** Summary statistics of the GHQ-28 score and its subscales among college students.

Subscales	Min	Max	Median	Mean	Standard Deviation
Somatic symptoms	0	21	5	5.64	4.16
Anxiety/insomnia	0	21	5	5.57	4.67
Social dysfunction	0	21	7	7.54	3.31
Severe depression	0	21	2	3.97	4.89
GHQ-28 total (0–84)	0	83	19	22.72	14.80

Table 3 shows the results of the Vuong test related to the fitting of different models, including Poisson regression, ZIP regression, NB regression, ZINB regression, GP regression, ZIGP regression, EEG regression and ZIEEG regression, to the daily number of cigarettes smoker by the college students. The results of the Vuong test were based on both BIC and AIC. According to the results of the Voung test statistics (both BIC and AIC), the Poisson regression (ZIP regression) provided the worst fit to the data among all regression models. Moreover, both Vuong test statistics did not show significant statistical differences between other methods. So, overall, the NB model was selected as the final model for simple interpretation.

**Table 3:** The results of the Vuong test for pairwise comparison of different distributions in modeling the daily number of cigarettes smoked among college students.

Vuong		Poisson	NB	GP	EEG	ZIP	ZINB	ZIGP
ZIEEG	AIC	6.331*	4.483*	3.0500*	4.431*	4.408*	−0.211	0.383
	BIC	5.581*	−1.742	−1.454	−1.947	4.316*	−0.211	0.383
ZIGP	AIC	6.295*	4.469*	3.100*	4.358*	4.236*	−0.477	
	BIC	5.548*	−1.828	−1.549	−2.010*	4.147*	−0.477	
ZINB	AIC	6.321*	4.564*	3.108*	4.499*	4.363*		
	BIC	5.572*	−1.757	−1.469	−1.960*	4.271*		
ZIP	AIC	5.835*	−4.484*	−4.137*	−4.626*			
	BIC	4.993*	−2.40*	−2.255*	−2.504*			
EEG	AIC	5.948*	1.197	−0.119				
	BIC	5.920*	1.197	−0.119				
GP	AIC	5.758*	0.321					
	BIC	5.731*	0.321					
NB	AIC	5.920*						
	BIC	5.892*						

*Significant at 0.05; negative binomial (NB), generalized Poisson (GP), exponentiated-exponential geometric (EEG) and their zero-inflated (ZI) counterparts.

Table 4 shows the regression coefficients of the NB regression model fitted to the daily number of cigarettes smoker by the college students. Exponentiated coefficients (incidence rate ratios (IRR)) and 95% confidence intervals were estimated for each model. According to the results shown in Table 4, the variables that were significantly associated with the daily number of cigarettes by the students included gender (male) (IRR = 9.45; 95% CI: 6.25, 14.28; P < 0.0001), Birth order (forth) (IRR = 2.05; 95% CI: 1.09, 3.90; P = 0.027), experiencing a break-up (IRR = 1.58; 95% CI: 1.05, 2.40; P = 0.027), having sexual intercourse (heterosexual vs. none: IRR = 2.59, 95% CI: 1.42 to 4.68, P = 0.002; homosexual vs. none: IRR = 3.13, 95% CI: 1.71 to 5.73, P < 0.001; homosexual vs. heterosexual: IRR = 1.21; 95% CI: 0.56 to 2.63, P = 0.628; having a history of drug abuse (opium/Psychedelic) (IRR = 5.99; 95% CI: 3.13, 11.51; P < 0.001).

**Table 4:** Regression coefficients obtained from the multivariate negative binomial regression model for modeling the daily number of cigarettes among college students.

Covariates	β (SE)	p-value	Exp(β)	95% Confidence Limits	
Intercept	−0.92	0.435	0.4	0.04	4.01
Gender					
Female	Reference category				
Male	2.25	<0.0001	9.45	6.25	14.28
Birth order					
First	Reference category				
Second	0.3	0.208	1.35	0.84	2.18
Third	0.03	0.901	1.03	0.6	1.77
Forth	0.72	0.027	2.05	1.09	3.9
Marital status					
Single/divorced	Reference category				
Married	−0.09	0.797	0.91	0.47	1.78
City					
Hometown (Hamadan)	Reference category				
Surrounding towns	−0.06	0.845	0.94	0.53	1.68
Towns of other provinces	−0.51	0.072	0.60	0.35	1.05
Residence					
Dormitory	Reference category				
Parents’ house	0.19	0.451	1.21	0.74	1.97
Student’s education level					
BSc	Reference category				
MSc	−0.06	0.881	0.94	0.45	1.98
MD	0.27	0.225	1.31	0.84	2.05
PhD	−0.77	0.212	0.46	0.14	1.55
The average grade of the previous semester	−0.01	0.935	0.99	0.88	1.12
Interested in the discipline					
Yes	Reference category				
No	0.51	0.079	1.67	0.94	2.97
Optimistic about future					
Yes	Reference category				
No	−0.19	0.529	0.83	0.45	1.5
Having a boyfriend/girlfriend					
No	Reference category				
Yes	0.39	0.079	1.48	0.96	2.27
Experiencing a break-up					
No	Reference category				
Yes	0.46	0.027	1.58	1.05	2.40
Having sexual intercourse					
None	Reference category				
Heterosexual	0.95	0.002	2.59	1.42	4.68
Homosexual	1.14	<0.0001	3.13	1.71	5.73
Drug abuse (opium/Psychedelic)					
No	Reference category				
Yes	1.79	<0.0001	5.99	3.13	11.51
Suicide thought lifetime					
No	Reference category				
Yes	0.03	0.919	1.03	0.53	2.03
Suicide attempt lifetime					
No	Reference category				
Yes	0.52	0.176	1.68	0.79	3.53
Using social media					
No	Reference category				
Yes	−0.28	0.424	0.76	0.38	1.5
GHQ28					
Not having psychiatric distress	Reference category				
Having psychiatric distress	0.001	0.994	1	0.66	1.52
C	5.01	<0.0001	—	—	—

## Discussion

Smoking intensity, defined usually as the number of cigarettes smoked by a person per day, can be considered as an important factor in establishing many serious smoking-related diseases, especially cancers. Smoking by college students, comprising a vast population of youth in Iran, makes them vulnerable to other risky behaviors. Therefore, investigating its underlying factors is of great importance. In this regard, count regression models are the first-line models that can be used to determine factors associated with smoking intensity as a count response, defined as the daily number of cigarettes smoked by an individual. There is no model that fits well for all data. So, selecting a model with the best fit to the data is of crucial importance. Here, the goodness-of-fit of several classical count regression models (Poisson, NB, GP, and EEG), as well as their zero-inflated counterparts (ZIP, ZINB, ZIGP, and ZIEEG), were investigated using a dataset related to the daily number of cigarettes smoked by college students. The findings of the present study revealed that the NB regression and Poisson regression had the best and worst fit to the data, respectively. Nevertheless, the goodness-of-fit of other models was comparable with that of the NB regression. So, the simplest model in terms of interpretation among them (NB regression) was selected as the most appropriate one. It is also possible to interpret the results of the ZINB regression model when one is interested in investigating factors associated with smoking/not-smoking and the number of cigarettes smoked per day as there may be different factors associated with each of them.

The findings of the present study indicated that having sexual intercourse increased the severity of smoking and led to smoking a greater number of cigarettes per day. Our findings revealed that having heterosexual and homosexual intercourse increased the daily number of cigarettes among the students by 2.59 and 3.13 times, respectively. While there might be few studies that investigate the association of these factors on the smoking severity as in this study, our findings were in concordance with the results of several studies that have investigated factors associated with smoker/nonsmoker response^41–46^. Moreover, according to our findings, homosexual students consumed a higher number of cigarettes per day compared to the heterosexual students (IRR = 1.21); however, it was not statistically significant, which may be due to the small number of homosexual and heterosexual students in the present study. Furthermore, due to the sensitive nature of questions about sexual activity in Iran, students may try to hide their sexual activities. Therefore, the fact that there may be some students in the “not having sexual intercourse” group that did not express their sexual orientation (heterosexual, gay/lesbian, and bisexual), because it is a social taboo, may attenuate the relationship of sexual orientation/activity with smoking among college students. There is a lot of evidence that tobacco use is higher among individuals identifying as lesbian, gay or bisexual (especially among women). Li *et al*. studied sex and sexual orientation in relation to tobacco use among young adult college students in the US^47^. They found that the pattern of tobacco use was different between heterosexual, gay/lesbian, and bisexual students; especially, bisexual women used a higher mean number of tobacco products compared to heterosexuals or other sexual minority groups. Hequembourg *et al*. also found that sexual minority women consumed more cigarettes smoked on smoking days compared to the heterosexual women^48^. The disparities in tobacco use across different sexual orientation groups have been reported by studies; so that a higher rate of tobacco use has been reported for bisexuals compared with gay/lesbian and heterosexuals and a higher rate of cigarette use has been reported for sexual minority versus heterosexual women^49–55^. In a study conducted by Zhang *et al*., it has been reported that homosexual people are more likely to engage in smoking^56^. Moreover, in another study conducted by Lindström *et al*., a higher smoking amount was observed for homosexual men compared with heterosexual men and women, while this quantity was not significant for homosexual women^57^. King and Nazareth found higher smoking rates for homosexual men and women compared with homosexual groups^58^. These evidences highlight the importance of targeting sexual minorities and considering the nuances across the sexual orientation spectrum in smoking cessation programs.

Our findings showed that illicit drug abuse, i.e. opium/psychedelic abuse as a high-risk behavior, was associated with consuming higher number of cigarettes per day among the students. Drug abusing has been also reported to be associated with a greater number of cigarettes smoked per day^59^. Having high-risk behaviors have been shown to be associated with psychiatric distress and suicidal ideation/attempt^60^. On the other hand, psychiatric distress and smoking have been shown to be associated positively^61,62^. It has been also reported that suicidal thoughts/attempts are strongly associated with smoking (OR = 4.03; 95% CI: 2.65–6.11)^60^.

Our findings also revealed that there was an association between experiencing a break-up and an increased daily number of cigarettes smoked by the students. The association between experiencing a break-up and smoking has not been investigated in the previous studies and this finding was novel. Experiencing a break-up might be related to the increased levels of psychosocial stress which is associated with greater odds of persistent smoking^63^.

Our findings also showed that the number of cigarettes smoked per day by male gender was higher than that of the female students by a factor of about 10. This finding is consistent with the findings of other studies. Moghimbeigi *et al*. in a study conducted in high schools in Iran showed that the daily number of cigarettes in male students was about 4 times greater than that of the female students^64^. Kilic and Ozturk in a study investigated the gender differences in cigarette consumption among adults in Turkey^65^. They found that the daily number of cigarettes in males was 1.6 times greater compared with the females. They also found that factors including education programs, cigarette taxation and tobacco advertising bans have different effects on each gender whereas social interaction is important for cigarette smoking behaviors of both genders. This might be attributed to the income elasticity among male students as they are more independent in terms of income than female students. Furthermore, it can be related to the differences in personality characteristics by gender. Traditional views also can cause differences in the social contacts of the students. While smoking by females is regarded as a taboo in the traditional culture of Iran, it is viewed as a common way of socializing with peers for males which might influence the smoking behavior for the male and female students^66^.

The findings of the present study indicated that the birth order was associated with the intensity of smoking, such that the greater number of cigarettes smoked per day was observed for higher orders of birth; and the daily number of cigarettes smoked by a student with the birth order of 4 was about 2 times greater compared with a student with the first order of birth. This finding was also consistent with the results of other studies^67^. Argys *et al*. found that “the number of cigarettes smoked daily increases monotonically with birth order, suggesting that the higher prevalence of smoking by later-borns found among U.S. adolescents”^68^. According to the theories, this may be attributed to the biological factors (changes in maternal immune system occurring over successive births), parents’ skills and experiences and having higher incomes during raising later-born children, such that parents treat the first child differently than the later-born children^69–74^.

As there were several sensitive questions in our used questionnaire, including those related to the sexual activities and drug consumption as well as the self-reported nature of the questionnaire, our results were limited due to the possibility of underestimation of the high-risk behaviors (the rejection rate was 6% among college students). One other limitation of this study was that questions about alcohol use were missed which is likely correlated with the outcome of interest and it is suggested to be considered in future studies. The cross-sectional nature of this study was another limitation that can limit our results; as the obtained results did not imply cause-effect relationships. Despite these limitations, we used multivariate methods to provide beneficial information about potential correlates of smoking intensity and tried to select a model that best fits the data among the most widely used count regression models.

The multivariate model utilized in the present research helped to identify correlates of smoking severity which should be taken into consideration while identifying smoking behavior among the students and establishing prevention and intervention programs for this population. In fact, these findings suggested that focusing on high-risk behaviors can be helpful in interventional programs for smoking cessation among college students.

## Supplementary information

Supplementary File 2

Supplementary File 1

## Data Availability

The dataset used and/or analyzed during the current study is available from the corresponding author on reasonable request.
