# Supplementary material for: Determining correlates of the average number of cigarette smoking among college students using count regression models
Source: Sci Rep. 2020 Jun 1;10:8874. doi: 10.1038/s41598-020-65813-4 (PMC7264191; doi:10.1038/s41598-020-65813-4)
Supplement: Supplementary file 1 — Supplementary File 2 [file 41598_2020_65813_MOESM1_ESM.pdf]

Title page

Determining correlates of the average number of cigarette smoking among college students using count regression models

Shararreh Parami

Department of Biostatistics, School of Public Health, Hamadan University of Medical Sciences, Hamadan, Iran

Email: sh.parami@edu.umsha.ac.ir

Leili Tapak

Department of Biostatistics, School of Public Health, Hamadan University of Medical Sciences, Hamadan, Iran

Modeling of Noncommunicable Diseases Research center, Hamadan University of Medical Sciences, Hamadan, Iran

Email: l.tapak@umsha.ac.ir

Abbas Moghimbeigi

Department of Biostatistics, School of Public health, Alborz University of Medical Sciences, Alborz, Iran

Email: moghimbeigi@umsha.ac.ir

Jalal Poorolajal

Department of Epidemiology, School of Public Health, Hamadan University of Medical Sciences, Hamadan, Iran

Research Center for Health Sciences, Hamadan University of Medical Sciences, Hamadan, Iran

Email: poorolajal@umsha.ac.ir

Ali Ghaleiha

Department of Psychiatry, School of Medicine, Hamadan University of Medical Sciences, Hamadan, Iran

Behavioral Disorders and Substance Abuse Research Center, Hamadan University of Medical Sciences, Hamadan, Iran

Email: Ghaleiha@umsha.ac.ir

Corresponding Author: Leili Tapak

Assistant Professor of Biostatistics, Department of Biostatistics, School of Public Health, Hamadan University of Medical Sciences, Hamadan, Iran

Email: l.tapak@umsha.ac.ir

Tel: 0098 918 110 3161

###Supplementary file 2

## Rcodes for voung test

```
##################################################################################GP
{
X<-read.csv("E:\\maghale1-98.4.8\\maghale1-data.csv")
X<-as.matrix(X) X
beta<-read.csv("E:\\maghale1-98.4.8\\maghale1-cofficients.csv")
beta<-na.omit(beta$GP)
beta<-as.matrix(beta)
beta1<-beta[1:26,]
beta1
alpha<-beta[27,]
number<-read.csv("E:\\maghale1-98.4.8\\number.csv")
Y<-as.matrix(number)
Y
y <- drop(Y)
ita<-X%*%beta1
ita
lambda<-drop(exp(ita))
lambda
ll.GP<- y*(log(lambda)-log(1+alpha*lambda))+(y-1)*log(1+alpha*y)-log(factorial(y))-
(lambda*(1+alpha*y))/(1+alpha*lambda)
total.ll.GP <- sum(ll.GP)
total.ll.GP
}#GP
#################################################################################EEGR
{
X<-read.csv("E:\\maghale1-98.4.8\\maghale1-data.csv")
X<-as.matrix(X)
beta<-read.csv("E:\\maghale1-98.4.8\\maghale1-cofficients.csv")
beta<-na.omit(beta$EEGR)
beta<-as.matrix(beta)
beta1<-beta[1:26,]
c<-beta[27,]
number<-read.csv("E:\\maghale1-98.4.8\\number.csv")
Y<-as.matrix(number)
Y
ita<-X%*%beta1
ita
lambda<- 1/(1+drop(exp(-ita)))
lambda
y <- drop(Y)
ll.EEGR<-log((1-lambda**(y+1))**c-(1-lambda**(y))**c)
total.ll.EEGR <- sum(ll.EEGR)
total.ll.EEGR
}#EEGR
################GP&EEGR
{
```

```
N<-1258
K1<-27
K2<-27
L1<-total.ll.GP
L2<-total.ll.EEGR
ll.1<-ll.GP
ll.2<-ll.EEGR
ll.t<- (ll.1)-(ll.2)
W2<- ((1/N)*sum((ll.t)^2))-((1/N)*sum(ll.t))^2
W<-sqrt(W2)
LR<-(L1-L2)-((K1-K2)/2)*log(N)
Z_statistic<-LR/(sqrt(N)*W)
Z_statistic
pnorm(Z_statistic)
Z_statistic<1.96
}#Z_statistic
################Vuong_AIC&Vuong_BIC
{
N<-1258
p2<-27
p1<-27
ll.2<-ll.GP
ll.1<-ll.EEGR
dli<- (ll.1)-(ll.2)
AIC_dlic<-dli+((p2-p1)/N)
BIC_dlic<-dli+((p2-p1)*log(N)/(2*N) )
SD<-sd(dli)
Vuong_AIC <- (SD * sqrt(N))^-1 * sum(AIC_dlic)
Vuong_AIC
Vuong_BIC <- (SD * sqrt(N))^-1 * sum(BIC_dlic)
Vuong_BIC
}#Vuong_AIC&Vuong_BIC
###########################################################################################GP
{
X<-read.csv("E:\\maghale1-98.4.8\\maghale1-data.csv")
X<-as.matrix(X)
X
beta<-read.csv("E:\\maghale1-98.4.8\\maghale1-cofficients.csv")
beta<-na.omit(beta$GP)
beta<-as.matrix(beta)
beta1<-beta[1:26,]
beta1
alpha<-beta[27,]
number<-read.csv("E:\\maghale1-98.4.8\\number.csv")
Y<-as.matrix(number)
Y
y <- drop(Y)
ita<-X%*%beta1
ita
```

```
lambda<-drop(exp(ita))
lambda
ll.GP<- y*(log(lambda)-log(1+alpha*lambda))+(y-1)*log(1+alpha*y)-log(factorial(y))-
(lambda*(1+alpha*y))/(1+alpha*lambda)
total.ll.GP <- sum(ll.GP)
total.ll.GP
}#GP
################################################################################ZIEEGR
{
X<-read.csv("E:\\maghale1-98.4.8\\maghale1-data.csv")
X<-as.matrix(X)
number<-read.csv("E:\\maghale1-98.4.8\\number.csv")
Y<-as.matrix(number)
Y
y <- drop(Y)
beta<-read.csv("E:\\maghale1-98.4.8\\maghale1-cofficients.csv")
ZIEEGR1<-na.omit(beta$ZIEEGR1)
ZIEEGR1<-as.matrix(ZIEEGR1)
beta1<-ZIEEGR1[1:26,]
c<-ZIEEGR1[27,]
c <- drop(c)
ita1<-X%*%beta1
ita1
lambda<-1/(1+drop(exp(-ita1)))
lambda
Z <- X
ZIEEGR2<-na.omit(beta$ZIEEGR2)
ZIEEGR2<-as.matrix(ZIEEGR2)
gamma1 <- ZIEEGR2
ita2<-Z%*%gamma1
ita2
P.loit <- 1/(1+drop(exp(-ita2)))
ll.count <- log(1-P.loit) + log((1-lambda^(y+1))^c-(1-lambda^(y))^c)
ll.zero <-log(P.loit + (1-P.loit)*(1-lambda)**c)
ll.ZIEEGR <- ifelse(y==0,ll.zero,ll.count)
total.ll.ZIEEGR<- sum(ll.ZIEEGR)
total.ll.ZIEEGR
}#ZIEEGR
################GP&ZIEEGR
{
N<-1258
K1<-27
K2<-53
L1<-total.ll.GP
L2<-total.ll.ZIEEGR
ll.1<-ll.GP
ll.2<-ll.ZIEEGR
ll.t<- (ll.1)-(ll.2)
W2<- ((1/N)*sum((ll.t)^2))-((1/N)*sum(ll.t))^2
```

```
W<-sqrt(W2)
LR<-(L1-L2)-((K1-K2)/2)*log(N)
Z_statistic<-LR/(sqrt(N)*W)
Z_statistic
pnorm(Z_statistic)
Z_statistic<1.96
}#Z_statistic
################Vuong_AIC&Vuong_BIC
{
N<-1258
p2<-27
p1<-53
ll.2<-ll.GP
ll.1<-ll.ZIEEGR
dli<- (ll.1)-(ll.2)
AIC_dlic<-dli+((p2-p1)/N)
BIC_dlic<-dli+((p2-p1)*log(N)/(2*N) )
SD<-sd(dli)
Vuong_AIC <- (SD * sqrt(N))^-1 * sum(AIC_dlic)
Vuong_AIC
Vuong_BIC <- (SD * sqrt(N))^-1 * sum(BIC_dlic)
Vuong_BIC
}#Vuong_AIC&Vuong_BIC
##########################################################################################GP
{
X<-read.csv("E:\\maghale1-98.4.8\\maghale1-data.csv")
X<-as.matrix(X)
X
beta<-read.csv("E:\\maghale1-98.4.8\\maghale1-cofficients.csv")
beta<-na.omit(beta$GP)
beta<-as.matrix(beta)
beta1<-beta[1:26,]
beta1
alpha<-beta[27,]
number<-read.csv("E:\\maghale1-98.4.8\\number.csv")
Y<-as.matrix(number)
Y
y <- drop(Y)
ita<-X%*%beta1
ita
lambda<-drop(exp(ita))
lambda
ll.GP<- y*(log(lambda)-log(1+alpha*lambda))+(y-1)*log(1+alpha*y)-log(factorial(y))-
(lambda*(1+alpha*y))/(1+alpha*lambda)
total.ll.GP <- sum(ll.GP)
total.ll.GP
}#GP
##########################################################################################ZIGP
{
```

```
X<-read.csv("E:\\maghale1-98.4.8\\maghale1-data.csv")
X<-as.matrix(X)
X
number<-read.csv("E:\\maghale1-98.4.8\\number.csv")
Y<-as.matrix(number)
Y
y <- drop(Y)
beta<-read.csv("E:\\maghale1-98.4.8\\maghale1-cofficients.csv")
ZIGP1<-na.omit(beta$ZIGP1)
ZIGP1<-as.matrix(ZIGP1)
beta1<-ZIGP1[1:26,]
alpha<-ZIGP1[27,]
ita1<-X%*%beta1
ita1
lambda<- drop(exp(ita1))
lambda
Z <- X
beta<-read.csv("E:\\maghale1-98.4.8\\maghale1-cofficients.csv")
ZIGP2<-na.omit(beta$ZIGP2)
ZIGP2<-as.matrix(ZIGP2)
gamma1 <- ZIGP2
ita2<-Z%*%gamma1
ita2
P.loit <- 1/(1+drop(exp(-ita2)))
ll.count <- log(1-P.loit) +y*(log(lambda)-log(1+alpha*lambda))+(y-1)*log(1+alpha*y)-log(factorial(y))-
(lambda*(1+ alpha*y))/(1+alpha*lambda)
ll.zero <-log(P.loit + (1-P.loit)*exp(-lambda/(1+alpha*lambda)))
ll.ZIGP <- ifelse(y==0,ll.zero,ll.count)
total.ll.ZIGP<- sum(ll.ZIGP)
total.ll.ZIGP
}#ZIGP
################GP&ZIGP
{
N<-1258
K1<-27
K2<-53
L1<-total.ll.GP
L2<-total.ll.ZIGP
ll.1<-ll.GP
ll.2<-ll.ZIGP
ll.t<- (ll.1)-(ll.2)
W2<- ((1/N)*sum((ll.t)^2))-((1/N)*sum(ll.t))^2
W<-sqrt(W2)
LR<-(L1-L2)-((K1-K2)/2)*log(N)
Z_statistic<-LR/(sqrt(N)*W)
Z_statistic
pnorm(Z_statistic)
Z_statistic<1.96
```

```
}#Z_statistic
################Vuong_AIC&Vuong_BIC
{
N<-1258
p2<-27
p1<-53
ll.2<-ll.GP
ll.1<-ll.ZIGP
dli<- (ll.1)-(ll.2)
AIC_dlic<-dli+((p2-p1)/N)
BIC_dlic<-dli+((p2-p1)*log(N)/(2*N) )
SD<-sd(dli)
Vuong_AIC <- (SD * sqrt(N))^-1 * sum(AIC_dlic)
Vuong_AIC
Vuong_BIC <- (SD * sqrt(N))^-1 * sum(BIC_dlic)
Vuong_BIC
}#Vuong_AIC&Vuong_BIC
########################################################################################GP
{
X<-read.csv("E:\\maghale1-98.4.8\\maghale1-data.csv")
X<-as.matrix(X)
X
beta<-read.csv("E:\\maghale1-98.4.8\\maghale1-cofficients.csv")
beta<-na.omit(beta$GP)
beta<-as.matrix(beta)
beta1<-beta[1:26,]
beta1
alpha<-beta[27,]
number<-read.csv("E:\\maghale1-98.4.8\\number.csv")
Y<-as.matrix(number)
Y
y <- drop(Y)
ita<-X%*%beta1
ita
lambda<-drop(exp(ita))
lambda
ll.GP<- y*(log(lambda)-log(1+alpha*lambda))+(y-1)*log(1+alpha*y)-log(factorial(y))-
(lambda*(1+alpha*y))/(1+alpha*lambda)
total.ll.GP <- sum(ll.GP)
total.ll.GP
}#GP
########################################################################################ZINB
{
X<-read.csv("E:\\maghale1-98.4.8\\maghale1-data.csv")
X<-as.matrix(X)
X
number<-read.csv("E:\\maghale1-98.4.8\\number.csv")
Y<-as.matrix(number)
Y
```

```
y <- drop(Y)
beta<-read.csv("E:\\maghale1-98.4.8\\maghale1-cofficients.csv")
ZINB1<-na.omit(beta$ZINB1)
ZINB1<-as.matrix(ZINB1)
beta1<-ZINB1[1:26,]
alpha<-ZINB1[27,]
ita1<-X%*%beta1
ita1
lambda<- drop(exp(ita1))
lambda
Z <- X
beta<-read.csv("E:\\maghale1-98.4.8\\maghale1-cofficients.csv")
ZINB2<-na.omit(beta$ZINB2)
ZINB2<-as.matrix(ZINB2)
gamma1 <- ZINB2
ita2<-Z%*%gamma1
ita2
m = 1/alpha
p = 1/(1+alpha*lambda)
P.loit <- 1/(1+drop(exp(-ita2)))
ll.count <- log(1-P.loit) + log(gamma(m + y)) - log(gamma(y + 1))- log(gamma(m)) + (m*log(p)) + (y*log(1-
p))
ll.zero <-log(P.loit + (1-P.loit)*(p**m))
ll.ZINB <- ifelse(y==0,ll.zero,ll.count)
total.ll.ZINB<-sum(ll.ZINB)
total.ll.ZINB
}#ZINB
################GP&ZINB
{
N<-1258
K1<-27
K2<-53
L1<-total.ll.GP
L2<-total.ll.ZINB
ll.1<-ll.GP
ll.2<-ll.ZINB
ll.t<- (ll.1)-(ll.2)
W2<- ((1/N)*sum((ll.t)^2))-((1/N)*sum(ll.t))^2
W<-sqrt(W2)
LR<-(L1-L2)-((K1-K2)/2)*log(N)
Z_statistic<-LR/(sqrt(N)*W)
Z_statistic
pnorm(Z_statistic)
Z_statistic<1.96
}#Z_statistic
################Vuong_AIC&Vuong_BIC
{
N<-1258
```

```
p2<-27
p1<-53
ll.2<-ll.GP
ll.1<-ll.ZINB
dli<- (ll.1)-(ll.2)
AIC_dlic<-dli+((p2-p1)/N)
BIC_dlic<-dli+((p2-p1)*log(N)/(2*N) )
SD<-sd(dli)
Vuong_AIC <- (SD * sqrt(N))^-1 * sum(AIC_dlic)
Vuong_AIC
Vuong_BIC <- (SD * sqrt(N))^-1 * sum(BIC_dlic)
Vuong_BIC
}#Vuong_AIC&Vuong_BIC
##########################################################################################GP
{
X<-read.csv("E:\\maghale1-98.4.8\\maghale1-data.csv")
X<-as.matrix(X)
X
beta<-read.csv("E:\\maghale1-98.4.8\\maghale1-cofficients.csv")
beta<-na.omit(beta$GP)
beta<-as.matrix(beta)
beta1<-beta[1:26,]
beta1
alpha<-beta[27,]
number<-read.csv("E:\\maghale1-98.4.8\\number.csv")
Y<-as.matrix(number)
Y
y <- drop(Y)
ita<-X%*%beta1
ita
lambda<-drop(exp(ita))
lambda
ll.GP<- y*(log(lambda)-log(1+alpha*lambda))+(y-1)*log(1+alpha*y)-log(factorial(y))-
(lambda*(1+alpha*y))/(1+alpha*lambda)
total.ll.GP <- sum(ll.GP)
total.ll.GP
}#GP
```

```
################################################################################ZIPoisson
{
X<-read.csv("E:\\maghale1-98.4.8\\maghale1-data.csv")
X<-as.matrix(X)
X
number<-read.csv("E:\\maghale1-98.4.8\\number.csv")
Y<-as.matrix(number)
Y
y <- drop(Y)
beta<-read.csv("E:\\maghale1-98.4.8\\maghale1-cofficients.csv")
ZIP1<-na.omit(beta$ZIP1)
ZIP1<-as.matrix(ZIP1)
ita1<-X%*%ZIP1
ita1
lambda<- drop(exp(ita1))
lambda
Z <- X
beta<-read.csv("E:\\maghale1-98.4.8\\maghale1-cofficients.csv")
ZIP2<-na.omit(beta$ZIP2)
ZIP2<-as.matrix(ZIP2)
gamma1 <- ZIP2
ita2<-Z%*%gamma1
ita2
P.loit <- 1/(1+drop(exp(-ita2)))
ll.count <- log((1-P.loit))-lambda +(y*log(lambda))-(log(factorial(y)))
ll.zero <-log(P.loit + (1-P.loit) * exp(-lambda))
ll.ZIPoisson <- ifelse(y==0,ll.zero,ll.count)
total.ll.ZIPoisson <- sum(ll.ZIPoisson)
total.ll.ZIPoisson
}#ZIPoisson
```

```
################GP&ZIP
{
N<-1258
K1<-27
K2<-52
L1<-total.ll.GP
L2<-total.ll.ZIPoisson
ll.1<-ll.GP
ll.2<-ll.ZIPoisson
ll.t<- (ll.1)-(ll.2)
W2<- ((1/N)*sum((ll.t)^2))-((1/N)*sum(ll.t))^2
W<-sqrt(W2)
LR<-(L1-L2)-((K1-K2)/2)*log(N)
Z_statistic<-LR/(sqrt(N)*W)
Z_statistic
pnorm(Z_statistic)
Z_statistic<1.96
}#Z_statistic
################Vuong_AIC&Vuong_BIC
{
N<-1258
p2<-27
p1<-52
ll.2<-ll.GP
ll.1<-ll.ZIPoisson
dli<- (ll.1)-(ll.2)
AIC_dlic<-dli+((p2-p1)/N)
BIC_dlic<-dli+((p2-p1)*log(N)/(2*N) )
SD<-sd(dli)
Vuong_AIC <- (SD * sqrt(N))^-1 * sum(AIC_dlic)
Vuong_AIC
Vuong_BIC <- (SD * sqrt(N))^-1 * sum(BIC_dlic)
Vuong_BIC
}#Vuong_AIC&Vuong_BIC
#######################################################################################EEGR
{
X<-read.csv("E:\\maghale1-98.4.8\\maghale1-data.csv")
X<-as.matrix(X)
beta<-read.csv("E:\\maghale1-98.4.8\\maghale1-cofficients.csv")
beta<-na.omit(beta$EEGR)
beta<-as.matrix(beta)
beta1<-beta[1:26,]
c<-beta[27,]
number<-read.csv("E:\\maghale1-98.4.8\\number.csv")
Y<-as.matrix(number)
Y
ita<-X%*%beta1
ita
lambda<- 1/(1+drop(exp(-ita)))
```

```
lambda
y <- drop(Y)
ll.EEGR<-log((1-lambda**(y+1))**c-(1-lambda**(y))**c)
total.ll.EEGR <- sum(ll.EEGR)
total.ll.EEGR
}#EEGR
#################################################################################ZIEEGR
{
X<-read.csv("E:\\maghale1-98.4.8\\maghale1-data.csv")
X<-as.matrix(X)
number<-read.csv("E:\\maghale1-98.4.8\\number.csv")
Y<-as.matrix(number)
Y
y <- drop(Y)
beta<-read.csv("E:\\maghale1-98.4.8\\maghale1-cofficients.csv")
ZIEEGR1<-na.omit(beta$ZIEEGR1)
ZIEEGR1<-as.matrix(ZIEEGR1)
beta1<-ZIEEGR1[1:26,]
c<-ZIEEGR1[27,]
c <- drop(c)
ita1<-X%*%beta1
ita1
lambda<-1/(1+drop(exp(-ita1)))
lambda
Z <- X
ZIEEGR2<-na.omit(beta$ZIEEGR2)
ZIEEGR2<-as.matrix(ZIEEGR2)
gamma1 <- ZIEEGR2
ita2<-Z%*%gamma1
ita2
P.loit <- 1/(1+drop(exp(-ita2)))
ll.count <- log(1-P.loit) + log((1-lambda^(y+1))^c-(1-lambda^(y))^c)
ll.zero <-log(P.loit + (1-P.loit)*(1-lambda)**c)
ll.ZIEEGR <- ifelse(y==0,ll.zero,ll.count)
total.ll.ZIEEGR<- sum(ll.ZIEEGR)
total.ll.ZIEEGR
```

```
}#ZIEEGR
################EEGR&ZIEEGR
{
N<-1258
K1<-27
K2<-53
L1<-total.ll.EEGR
L2<-total.ll.ZIEEGR
ll.1<-ll.EEGR
ll.2<-ll.ZIEEGR
ll.t<- (ll.1)-(ll.2)
W2<- ((1/N)*sum((ll.t)^2))-((1/N)*sum(ll.t))^2
W<-sqrt(W2)
LR<-(L1-L2)-((K1-K2)/2)*log(N)
Z_statistic<-LR/(sqrt(N)*W)
Z_statistic
pnorm(Z_statistic)
Z_statistic<1.96
}#Z_statistic
################Vuong_AIC&Vuong_BIC
{
N<-1258
p2<-27
p1<-53
ll.2<-ll.EEGR
ll.1<-ll.ZIEEGR
dli<- (ll.1)-(ll.2)
AIC_dlic<-dli+((p2-p1)/N)
BIC_dlic<-dli+((p2-p1)*log(N)/(2*N) )
SD<-sd(dli)
Vuong_AIC <- (SD * sqrt(N))^-1 * sum(AIC_dlic)
Vuong_AIC
Vuong_BIC <- (SD * sqrt(N))^-1 * sum(BIC_dlic)
Vuong_BIC
}#Vuong_AIC&Vuong_BIC
###########################################################################################EEGR
{
X<-read.csv("E:\\maghale1-98.4.8\\maghale1-data.csv")
X<-as.matrix(X)
beta<-read.csv("E:\\maghale1-98.4.8\\maghale1-cofficients.csv")
beta<-na.omit(beta$EEGR)
beta<-as.matrix(beta)
beta1<-beta[1:26,]
c<-beta[27,]
number<-read.csv("E:\\maghale1-98.4.8\\number.csv")
Y<-as.matrix(number)
Y
ita<-X%*%beta1
ita
```

```
lambda<- 1/(1+drop(exp(-ita)))
lambda
y <- drop(Y)
ll.EEGR<-log((1-lambda**(y+1))**c-(1-lambda**(y))**c)
total.ll.EEGR <- sum(ll.EEGR)
total.ll.EEGR
}#EEGR
#################################################################################ZIGP
{
X<-read.csv("E:\\maghale1-98.4.8\\maghale1-data.csv")
X<-as.matrix(X)
X
number<-read.csv("E:\\maghale1-98.4.8\\number.csv")
Y<-as.matrix(number)
Y
y <- drop(Y)
beta<-read.csv("E:\\maghale1-98.4.8\\maghale1-cofficients.csv")
ZIGP1<-na.omit(beta$ZIGP1)
ZIGP1<-as.matrix(ZIGP1)
beta1<-ZIGP1[1:26,]
alpha<-ZIGP1[27,]
ita1<-X%*%beta1
ita1
lambda<- drop(exp(ita1))
lambda
Z <- X
beta<-read.csv("E:\\maghale1-98.4.8\\maghale1-cofficients.csv")
ZIGP2<-na.omit(beta$ZIGP2)
ZIGP2<-as.matrix(ZIGP2)
gamma1 <- ZIGP2
ita2<-Z%*%gamma1
ita2
P.loit <- 1/(1+drop(exp(-ita2)))
ll.count <- log(1-P.loit) +y*(log(lambda)-log(1+alpha*lambda))+(y-1)*log(1+alpha*y)-log(factorial(y))-
(lambda*(1+ alpha*y))/(1+alpha*lambda)
ll.zero <-log(P.loit + (1-P.loit)*exp(-lambda/(1+alpha*lambda)))
ll.ZIGP <- ifelse(y==0,ll.zero,ll.count)
total.ll.ZIGP<- sum(ll.ZIGP)
```

```
total.ll.ZIGP
}#ZIGP
################EEGR&ZIGP
{
N<-1258
K1<-27
K2<-53
L1<-total.ll.EEGR
L2<-total.ll.ZIGP
ll.1<-ll.EEGR
ll.2<-ll.ZIGP
ll.t<- (ll.1)-(ll.2)
W2<- ((1/N)*sum((ll.t)^2))-((1/N)*sum(ll.t))^2
W<-sqrt(W2)
LR<-(L1-L2)-((K1-K2)/2)*log(N)
Z_statistic<-LR/(sqrt(N)*W)
Z_statistic
pnorm(Z_statistic)
Z_statistic<1.96
}#Z_statistic
################Vuong_AIC&Vuong_BIC
{
N<-1258
p2<-27
p1<-53
ll.2<-ll.EEGR
ll.1<-ll.ZIGP
dli<- (ll.1)-(ll.2)
AIC_dlic<-dli+((p2-p1)/N)
BIC_dlic<-dli+((p2-p1)*log(N)/(2*N) )
SD<-sd(dli)
Vuong_AIC <- (SD * sqrt(N))^-1 * sum(AIC_dlic)
Vuong_AIC
Vuong_BIC <- (SD * sqrt(N))^-1 * sum(BIC_dlic)
Vuong_BIC
}#Vuong_AIC&Vuong_BIC
#####################################################################################EEGR
{
X<-read.csv("E:\\maghale1-98.4.8\\maghale1-data.csv")
X<-as.matrix(X)
beta<-read.csv("E:\\maghale1-98.4.8\\maghale1-cofficients.csv")
beta<-na.omit(beta$EEGR)
beta<-as.matrix(beta)
beta1<-beta[1:26,]
c<-beta[27,]
number<-read.csv("E:\\maghale1-98.4.8\\number.csv")
Y<-as.matrix(number)
Y
ita<-X%*%beta1
```

```
ita
lambda<- 1/(1+drop(exp(-ita)))
lambda
y <- drop(Y)
ll.EEGR<-log((1-lambda**(y+1))**c-(1-lambda**(y))**c)
total.ll.EEGR <- sum(ll.EEGR)
total.ll.EEGR
}#EEGR
#####################################################################################ZINB
{
X<-read.csv("E:\\maghale1-98.4.8\\maghale1-data.csv")
X<-as.matrix(X)
X
number<-read.csv("E:\\maghale1-98.4.8\\number.csv")
Y<-as.matrix(number)
Y
y <- drop(Y)
beta<-read.csv("E:\\maghale1-98.4.8\\maghale1-cofficients.csv")
ZINB1<-na.omit(beta$ZINB1)
ZINB1<-as.matrix(ZINB1)
beta1<-ZINB1[1:26,]
alpha<-ZINB1[27,]
ita1<-X%*%beta1
ita1
lambda<- drop(exp(ita1))
lambda
Z <- X
beta<-read.csv("E:\\maghale1-98.4.8\\maghale1-cofficients.csv")
ZINB2<-na.omit(beta$ZINB2)
ZINB2<-as.matrix(ZINB2)
gamma1 <- ZINB2
ita2<-Z%*%gamma1
ita2
m = 1/alpha
p = 1/(1+alpha*lambda)
P.loit <- 1/(1+drop(exp(-ita2)))
ll.count <- log(1-P.loit) + log(gamma(m + y)) - log(gamma(y + 1))- log(gamma(m)) + (m*log(p)) + (y*log(1-
p))
ll.zero <-log(P.loit + (1-P.loit)*(p**m))
ll.ZINB <- ifelse(y==0,ll.zero,ll.count)
total.ll.ZINB<-sum(ll.ZINB)
total.ll.ZINB
}#ZINB
################EEGR&ZINB
{
N<-1258
K1<-27
K2<-53
L1<-total.ll.EEGR
```

```
L2<-total.ll.ZINB
ll.1<-ll.EEGR
ll.2<-ll.ZINB
ll.t<- (ll.1)-(ll.2)
W2<- ((1/N)*sum((ll.t)^2))-((1/N)*sum(ll.t))^2
W<-sqrt(W2)
LR<-(L1-L2)-((K1-K2)/2)*log(N)
Z_statistic<-LR/(sqrt(N)*W)
Z_statistic
pnorm(Z_statistic)
Z_statistic<1.96
}#Z_statistic
################Vuong_AIC&Vuong_BIC
{
N<-1258
p2<-27
p1<-53
ll.2<-ll.EEGR
ll.1<-ll.ZINB
dli<- (ll.1)-(ll.2)
AIC_dlic<-dli+((p2-p1)/N)
BIC_dlic<-dli+((p2-p1)*log(N)/(2*N) )
SD<-sd(dli)
Vuong_AIC <- (SD * sqrt(N))^-1 * sum(AIC_dlic)
Vuong_AIC
Vuong_BIC <- (SD * sqrt(N))^-1 * sum(BIC_dlic)
Vuong_BIC
}#Vuong_AIC&Vuong_BIC
##########################################################################################EEGR
{
X<-read.csv("E:\\maghale1-98.4.8\\maghale1-data.csv")
X<-as.matrix(X)
beta<-read.csv("E:\\maghale1-98.4.8\\maghale1-cofficients.csv")
beta<-na.omit(beta$EEGR)
beta<-as.matrix(beta)
beta1<-beta[1:26,]
c<-beta[27,]
number<-read.csv("E:\\maghale1-98.4.8\\number.csv")
Y<-as.matrix(number)
Y
ita<-X%*%beta1
ita
lambda<- 1/(1+drop(exp(-ita)))
lambda
y <- drop(Y)
ll.EEGR<-log((1-lambda**(y+1))**c-(1-lambda**(y))**c)
total.ll.EEGR <- sum(ll.EEGR)
```

```
total.ll.EEGR
}#EEGR
#####################################################################################ZIPoisson
{
X<-read.csv("E:\\maghale1-98.4.8\\maghale1-data.csv")
X<-as.matrix(X)
X
number<-read.csv("E:\\maghale1-98.4.8\\number.csv")
Y<-as.matrix(number)
Y
y <- drop(Y)
beta<-read.csv("E:\\maghale1-98.4.8\\maghale1-cofficients.csv")
ZIP1<-na.omit(beta$ZIP1)
ZIP1<-as.matrix(ZIP1)
ita1<-X%*%ZIP1
ita1
lambda<- drop(exp(ita1))
lambda
Z <- X
beta<-read.csv("E:\\maghale1-98.4.8\\maghale1-cofficients.csv")
ZIP2<-na.omit(beta$ZIP2)
ZIP2<-as.matrix(ZIP2)
gamma1 <- ZIP2
ita2<-Z%*%gamma1
ita2
P.loit <- 1/(1+drop(exp(-ita2)))
ll.count <- log((1-P.loit))-lambda +(y*log(lambda))-(log(factorial(y)))
ll.zero <-log(P.loit + (1-P.loit) * exp(-lambda))
ll.ZIPoisson <- ifelse(y==0,ll.zero,ll.count)
total.ll.ZIPoisson <- sum(ll.ZIPoisson)
total.ll.ZIPoisson
}#ZIPoisson
################EEGR&ZIP
{
N<-1258
K1<-27
K2<-52
L1<-total.ll.EEGR
L2<-total.ll.ZIPoisson
ll.1<-ll.EEGR
ll.2<-ll.ZIPoisson
ll.t<- (ll.1)-(ll.2)
W2<- ((1/N)*sum((ll.t)^2))-((1/N)*sum(ll.t))^2
W<-sqrt(W2)
LR<-(L1-L2)-((K1-K2)/2)*log(N)
Z_statistic<-LR/(sqrt(N)*W)
Z_statistic
pnorm(Z_statistic)
```

```
Z_statistic<1.96
}#Z_statistic
################Vuong_AIC&Vuong_BIC
{
N<-1258
p2<-27
p1<-52
ll.2<-ll.EEGR
ll.1<-ll.ZIPoisson
dli<- (ll.1)-(ll.2)
AIC_dlic<-dli+((p2-p1)/N)
BIC_dlic<-dli+((p2-p1)*log(N)/(2*N) )
SD<-sd(dli)
Vuong_AIC <- (SD * sqrt(N))^-1 * sum(AIC_dlic)
Vuong_AIC
Vuong_BIC <- (SD * sqrt(N))^-1 * sum(BIC_dlic)
Vuong_BIC
}#Vuong_AIC&Vuong_BIC
#########################################################################################NB
{
X<-read.csv("E:\\maghale1-98.4.8\\maghale1-data.csv")
X<-as.matrix(X)
X
class(X)
beta<-read.csv("E:\\maghale1-98.4.8\\maghale1-cofficients.csv")
beta<-na.omit(beta$NB)
beta<-as.matrix(beta)
beta1<-beta[1:26,]
beta1
number<-read.csv("E:\\maghale1-98.4.8\\number.csv")
Y<-as.matrix(number)
Y
ita<-X%*%beta1
ita
lambda<- drop(exp(ita))
lambda
y <- drop(Y)
alpha<-beta[27,]
m = 1/alpha
```

```
p = 1/(1+alpha*lambda)
ll.NB<-log(gamma(m + y)) - log(gamma(y + 1))- log(gamma(m)) + (m*log(p)) + y*log(1-p)
total.ll.NB <- sum(ll.NB)
total.ll.NB
}#NB
######################################################################################EEGR
{
X<-read.csv("E:\\maghale1-98.4.8\\maghale1-data.csv")
X<-as.matrix(X)
beta<-read.csv("E:\\maghale1-98.4.8\\maghale1-cofficients.csv")
beta<-na.omit(beta$EEGR)
beta<-as.matrix(beta)
beta1<-beta[1:26,]
c<-beta[27,]
number<-read.csv("E:\\maghale1-98.4.8\\number.csv")
Y<-as.matrix(number)
Y
ita<-X%*%beta1
ita
lambda<- 1/(1+drop(exp(-ita)))
lambda
y <- drop(Y)
ll.EEGR<-log((1-lambda**(y+1))**c-(1-lambda**(y))**c)
total.ll.EEGR <- sum(ll.EEGR)
total.ll.EEGR
}#EEGR
################NB&EEGR
{
N<-1258
K1<-27
K2<-27
L1<-total.ll.NB
L2<-total.ll.EEGR
ll.1<-ll.NB
ll.2<-ll.EEGR
ll.t<- (ll.1)-(ll.2)
W2<- ((1/N)*sum((ll.t)^2))-((1/N)*sum(ll.t))^2
W<-sqrt(W2)
LR<-(L1-L2)-((K1-K2)/2)*log(N)
Z_statistic<-LR/(sqrt(N)*W)
Z_statistic
pnorm(Z_statistic)
Z_statistic<1.96
}#Z_statistic
################Vuong_AIC&Vuong_BIC
{
N<-1258
p2<-27
p1<-27
```

```
ll.2<-ll.NB
ll.1<-ll.EEGR
dli<- (ll.1)-(ll.2)
AIC_dlic<-dli+((p2-p1)/N)
BIC_dlic<-dli+((p2-p1)*log(N)/(2*N) )
SD<-sd(dli)
Vuong_AIC <- (SD * sqrt(N))^-1 * sum(AIC_dlic)
Vuong_AIC
Vuong_BIC <- (SD * sqrt(N))^-1 * sum(BIC_dlic)
Vuong_BIC
}#Vuong_AIC&Vuong_BIC
####################################################################################NB
{
X<-read.csv("E:\\maghale1-98.4.8\\maghale1-data.csv")
X<-as.matrix(X)
X
class(X)
beta<-read.csv("E:\\maghale1-98.4.8\\maghale1-cofficients.csv")
beta<-na.omit(beta$NB)
beta<-as.matrix(beta)
beta1<-beta[1:26,]
beta1
number<-read.csv("E:\\maghale1-98.4.8\\number.csv")
Y<-as.matrix(number)
Y
ita<-X%*%beta1
ita
lambda<- drop(exp(ita))
lambda
y <- drop(Y)
alpha<-beta[27,]
m = 1/alpha
p = 1/(1+alpha*lambda)
ll.NB<-log(gamma(m + y)) - log(gamma(y + 1))- log(gamma(m)) + (m*log(p)) + y*log(1-p)
total.ll.NB <- sum(ll.NB)
total.ll.NB
}#NB
```

```
####################################################################################ZIGP
{
X<-read.csv("E:\\maghale1-98.4.8\\maghale1-data.csv")
X<-as.matrix(X)
X
beta<-read.csv("E:\\maghale1-98.4.8\\maghale1-cofficients.csv")
beta<-na.omit(beta$GP)
beta<-as.matrix(beta)
beta1<-beta[1:26,]
beta1
alpha<-beta[27,]
number<-read.csv("E:\\maghale1-98.4.8\\number.csv")
Y<-as.matrix(number)
Y
y <- drop(Y)
ita<-X%*%beta1
ita
lambda<-drop(exp(ita))
lambda
ll.GP<- y*(log(lambda)-log(1+alpha*lambda))+(y-1)*log(1+alpha*y)-log(factorial(y))-
(lambda*(1+alpha*y))/(1+alpha*lambda)
total.ll.GP <- sum(ll.GP)
total.ll.GP
}#GP
################NB&GP
{
N<-1258
K1<-27
K2<-27
L1<-total.ll.NB
L2<-total.ll.GP
ll.1<-ll.NB
ll.2<-ll.GP
ll.t<- (ll.1)-(ll.2)
W2<- ((1/N)*sum((ll.t)^2))-((1/N)*sum(ll.t))^2
W<-sqrt(W2)
LR<-(L1-L2)-((K1-K2)/2)*log(N)
Z_statistic<-LR/(sqrt(N)*W)
Z_statistic
pnorm(Z_statistic)
Z_statistic<1.96
}#Z_statistic
################Vuong_AIC&Vuong_BIC
{
N<-1258
p2<-27
p1<-27
ll.2<-ll.NB
ll.1<-ll.GP
```

```
dli<- (ll.1)-(ll.2)
AIC_dlic<-dli+((p2-p1)/N)
BIC_dlic<-dli+((p2-p1)*log(N)/(2*N) )
SD<-sd(dli)
Vuong_AIC <- (SD * sqrt(N))^-1 * sum(AIC_dlic)
Vuong_AIC
Vuong_BIC <- (SD * sqrt(N))^-1 * sum(BIC_dlic)
Vuong_BIC
}#Vuong_AIC&Vuong_BIC
###########################################################################################NB
{
X<-read.csv("E:\\maghale1-98.4.8\\maghale1-data.csv")
X<-as.matrix(X)
X
class(X)
beta<-read.csv("E:\\maghale1-98.4.8\\maghale1-cofficients.csv")
beta<-na.omit(beta$NB)
beta<-as.matrix(beta)
beta1<-beta[1:26,]
beta1
number<-read.csv("E:\\maghale1-98.4.8\\number.csv")
Y<-as.matrix(number)
Y
ita<-X%*%beta1
ita
lambda<- drop(exp(ita))
lambda
y <- drop(Y)
alpha<-beta[27,]
m = 1/alpha
p = 1/(1+alpha*lambda)
ll.NB<-log(gamma(m + y)) - log(gamma(y + 1))- log(gamma(m)) + (m*log(p)) + y*log(1-p)
total.ll.NB <- sum(ll.NB)
total.ll.NB
}#NB
#####################################################################################ZIEEGR
{
```

```
X<-read.csv("E:\\maghale1-98.4.8\\maghale1-data.csv")
X<-as.matrix(X)
number<-read.csv("E:\\maghale1-98.4.8\\number.csv")
Y<-as.matrix(number)
Y
y <- drop(Y)
beta<-read.csv("E:\\maghale1-98.4.8\\maghale1-cofficients.csv")
ZIEEGR1<-na.omit(beta$ZIEEGR1)
ZIEEGR1<-as.matrix(ZIEEGR1)
beta1<-ZIEEGR1[1:26,]
c<-ZIEEGR1[27,]
c <- drop(c)
ita1<-X%*%beta1
ita1
lambda<-1/(1+drop(exp(-ita1)))
lambda
Z <- X
ZIEEGR2<-na.omit(beta$ZIEEGR2)
ZIEEGR2<-as.matrix(ZIEEGR2)
gamma1 <- ZIEEGR2
ita2<-Z%*%gamma1
ita2
P.loit <- 1/(1+drop(exp(-ita2)))
ll.count <- log(1-P.loit) + log((1-lambda^(y+1))^c-(1-lambda^(y))^c)
ll.zero <-log(P.loit + (1-P.loit)*(1-lambda)**c)
ll.ZIEEGR <- ifelse(y==0,ll.zero,ll.count)
total.ll.ZIEEGR<- sum(ll.ZIEEGR)
total.ll.ZIEEGR
}#ZIEEGR
################NB&ZIEEGR
{
N<-1258
K1<-27
K2<-53
L1<-total.ll.NB
L2<-total.ll.ZIEEGR
ll.1<-ll.NB
ll.2<-ll.ZIEEGR
ll.t<- (ll.1)-(ll.2)
W2<- ((1/N)*sum((ll.t)^2))-((1/N)*sum(ll.t))^2
W<-sqrt(W2)
LR<-(L1-L2)-((K1-K2)/2)*log(N)
Z_statistic<-LR/(sqrt(N)*W)
Z_statistic
pnorm(Z_statistic)
Z_statistic<1.96
}#Z_statistic
################Vuong_AIC&Vuong_BIC
```

```
{
N<-1258
p2<-27
p1<-53
ll.2<-ll.NB
ll.1<-ll.ZIEEGR
dli<- (ll.1)-(ll.2)
AIC_dlic<-dli+((p2-p1)/N)
BIC_dlic<-dli+((p2-p1)*log(N)/(2*N) )
SD<-sd(dli)  Vuong_AIC <- (SD * sqrt(N))^-1 * sum(AIC_dlic)
Vuong_AIC
Vuong_BIC <- (SD * sqrt(N))^-1 * sum(BIC_dlic)
Vuong_BIC
}#Vuong_AIC&Vuong_BIC
########################################################################################NB
{
X<-read.csv("E:\\maghale1-98.4.8\\maghale1-data.csv")
X<-as.matrix(X)
X
class(X)
beta<-read.csv("E:\\maghale1-98.4.8\\maghale1-cofficients.csv")
beta<-na.omit(beta$NB)
beta<-as.matrix(beta)
beta1<-beta[1:26,]
beta1
number<-read.csv("E:\\maghale1-98.4.8\\number.csv")
Y<-as.matrix(number)
Y
ita<-X%*%beta1
ita
lambda<- drop(exp(ita))
lambda
y <- drop(Y)
alpha<-beta[27,]
m = 1/alpha
p = 1/(1+alpha*lambda)
ll.NB<-log(gamma(m + y)) - log(gamma(y + 1))- log(gamma(m)) + (m*log(p)) + y*log(1-p)
total.ll.NB <- sum(ll.NB)
total.ll.NB
}#NB
#######################################################################################ZIGP
{
X<-read.csv("E:\\maghale1-98.4.8\\maghale1-data.csv")
X<-as.matrix(X)
X
number<-read.csv("E:\\maghale1-98.4.8\\number.csv")
Y<-as.matrix(number)
Y
y <- drop(Y)
```

```
beta<-read.csv("E:\\maghale1-98.4.8\\maghale1-cofficients.csv")
ZIGP1<-na.omit(beta$ZIGP1)
ZIGP1<-as.matrix(ZIGP1)
beta1<-ZIGP1[1:26,]
alpha<-ZIGP1[27,]
ita1<-X%*%beta1
ita1
lambda<- drop(exp(ita1))
lambda
Z <- X
beta<-read.csv("E:\\maghale1-98.4.8\\maghale1-cofficients.csv")
ZIGP2<-na.omit(beta$ZIGP2)
ZIGP2<-as.matrix(ZIGP2)
gamma1 <- ZIGP2
ita2<-Z%*%gamma1
ita2
P.loit <- 1/(1+drop(exp(-ita2)))
ll.count <- log(1-P.loit) +y*(log(lambda)-log(1+alpha*lambda))+(y-1)*log(1+alpha*y)-log(factorial(y))-
(lambda*(1+ alpha*y))/(1+alpha*lambda)
ll.zero <-log(P.loit + (1-P.loit)*exp(-lambda/(1+alpha*lambda)))
ll.ZIGP <- ifelse(y==0,ll.zero,ll.count)
total.ll.ZIGP<- sum(ll.ZIGP)
total.ll.ZIGP
}#ZIGP
################NB&ZIGP
{
N<-1258
K1<-27
K2<-53
L1<-total.ll.NB
L2<-total.ll.ZIGP
ll.1<-ll.NB
ll.2<-ll.ZIGP
ll.t<- (ll.1)-(ll.2)
W2<- ((1/N)*sum((ll.t)^2))-((1/N)*sum(ll.t))^2
W<-sqrt(W2)
LR<-(L1-L2)-((K1-K2)/2)*log(N)
Z_statistic<-LR/(sqrt(N)*W)
Z_statistic
pnorm(Z_statistic)
Z_statistic<1.96
}#Z_statistic
################Vuong_AIC&Vuong_BIC
{
N<-1258
p2<-27
```

```
p1<-53
ll.2<-ll.NB
ll.1<-ll.ZIGP
dli<- (ll.1)-(ll.2)
AIC_dlic<-dli+((p2-p1)/N)
BIC_dlic<-dli+((p2-p1)*log(N)/(2*N) )
SD<-sd(dli)
Vuong_AIC <- (SD * sqrt(N))^-1 * sum(AIC_dlic)
Vuong_AIC
Vuong_BIC <- (SD * sqrt(N))^-1 * sum(BIC_dlic)
Vuong_BIC
}#Vuong_AIC&Vuong_BIC
##########################################################################################NB
{
X<-read.csv("E:\\maghale1-98.4.8\\maghale1-data.csv")
X<-as.matrix(X)
X
class(X)
beta<-read.csv("E:\\maghale1-98.4.8\\maghale1-cofficients.csv")
beta<-na.omit(beta$NB)
beta<-as.matrix(beta)
beta1<-beta[1:26,]
beta1
number<-read.csv("E:\\maghale1-98.4.8\\number.csv")
Y<-as.matrix(number)
Y
ita<-X%*%beta1
ita
lambda<- drop(exp(ita))
lambda
y <- drop(Y)
alpha<-beta[27,]
m = 1/alpha
p = 1/(1+alpha*lambda)
ll.NB<-log(gamma(m + y)) - log(gamma(y + 1))- log(gamma(m)) + (m*log(p)) + y*log(1-p)
total.ll.NB <- sum(ll.NB)
total.ll.NB
}#NB
##########################################################################################ZINB
{
X<-read.csv("E:\\maghale1-98.4.8\\maghale1-data.csv")
X<-as.matrix(X)
X
number<-read.csv("E:\\maghale1-98.4.8\\number.csv")
Y<-as.matrix(number)
Y
y <- drop(Y)
beta<-read.csv("E:\\maghale1-98.4.8\\maghale1-cofficients.csv")
ZINB1<-na.omit(beta$ZINB1)
```

```
ZINB1<-as.matrix(ZINB1)
beta1<-ZINB1[1:26,]
alpha<-ZINB1[27,]
ita1<-X%*%beta1
ita1
lambda<- drop(exp(ita1))
lambda
Z <- X
beta<-read.csv("E:\\maghale1-98.4.8\\maghale1-cofficients.csv")
ZINB2<-na.omit(beta$ZINB2)
ZINB2<-as.matrix(ZINB2)
gamma1 <- ZINB2
ita2<-Z%*%gamma1
ita2
m = 1/alpha
p = 1/(1+alpha*lambda)
P.loit <- 1/(1+drop(exp(-ita2)))
ll.count <- log(1-P.loit) + log(gamma(m + y)) - log(gamma(y + 1))- log(gamma(m)) + (m*log(p)) + (y*log(1-
p))
ll.zero <-log(P.loit + (1-P.loit)*(p**m))
ll.ZINB <- ifelse(y==0,ll.zero,ll.count)
total.ll.ZINB<-sum(ll.ZINB)
total.ll.ZINB
}#ZINB
################NB&ZINB
{
N<-1258
K1<-27
K2<-53
L1<-total.ll.NB
L2<-total.ll.ZINB
ll.1<-ll.NB
ll.2<-ll.ZINB
ll.t<- (ll.1)-(ll.2)
W2<- ((1/N)*sum((ll.t)^2))-((1/N)*sum(ll.t))^2
W<-sqrt(W2)
LR<-(L1-L2)-((K1-K2)/2)*log(N)
Z_statistic<-LR/(sqrt(N)*W)
Z_statistic
pnorm(Z_statistic)
Z_statistic<1.96
}#Z_statistic
################Vuong_AIC&Vuong_BIC
{
N<-1258
p2<-27
p1<-53
ll.2<-ll.NB
```

```
ll.1<-ll.ZINB
dli<- (ll.1)-(ll.2)
AIC_dlic<-dli+((p2-p1)/N)
BIC_dlic<-dli+((p2-p1)*log(N)/(2*N) )
SD<-sd(dli)
Vuong_AIC <- (SD * sqrt(N))^-1 * sum(AIC_dlic)
Vuong_AIC
Vuong_BIC <- (SD * sqrt(N))^-1 * sum(BIC_dlic)
Vuong_BIC
}#Vuong_AIC&Vuong_BIC
####################################################################################NB
{
X<-read.csv("E:\\maghale1-98.4.8\\maghale1-data.csv")
X<-as.matrix(X)
X
class(X)
beta<-read.csv("E:\\maghale1-98.4.8\\maghale1-cofficients.csv")
beta<-na.omit(beta$NB)
beta<-as.matrix(beta)
beta1<-beta[1:26,]
beta1
number<-read.csv("E:\\maghale1-98.4.8\\number.csv")
Y<-as.matrix(number)
Y
ita<-X%*%beta1
ita
lambda<- drop(exp(ita))
lambda
y <- drop(Y)
alpha<-beta[27,]
m = 1/alpha
p = 1/(1+alpha*lambda)
ll.NB<-log(gamma(m + y)) - log(gamma(y + 1))- log(gamma(m)) + (m*log(p)) + y*log(1-p)
total.ll.NB <- sum(ll.NB)
total.ll.NB
}#NB
#################################################################################ZIPoisson
{
X<-read.csv("E:\\maghale1-98.4.8\\maghale1-data.csv")
X<-as.matrix(X)
X
number<-read.csv("E:\\maghale1-98.4.8\\number.csv")
Y<-as.matrix(number)
Y
y <- drop(Y)
beta<-read.csv("E:\\maghale1-98.4.8\\maghale1-cofficients.csv")
```

```
ZIP1<-na.omit(beta$ZIP1)
ZIP1<-as.matrix(ZIP1)
ita1<-X%*%ZIP1
ita1
lambda<- drop(exp(ita1))
lambda
Z <- X
beta<-read.csv("E:\\maghale1-98.4.8\\maghale1-cofficients.csv")
ZIP2<-na.omit(beta$ZIP2)
ZIP2<-as.matrix(ZIP2)
gamma1 <- ZIP2
ita2<-Z%*%gamma1
ita2
P.loit <- 1/(1+drop(exp(-ita2)))
ll.count <- log((1-P.loit))-lambda +(y*log(lambda))-(log(factorial(y)))
ll.zero <-log(P.loit + (1-P.loit) * exp(-lambda))
ll.ZIPoisson <- ifelse(y==0,ll.zero,ll.count)
total.ll.ZIPoisson <- sum(ll.ZIPoisson)
total.ll.ZIPoisson
}#ZIPoisson
################NB&ZIP
{
N<-1258
K1<-27
K2<-52
L1<-total.ll.NB
L2<-total.ll.ZIPoisson
ll.1<-ll.NB
ll.2<-ll.ZIPoisson
ll.t<- (ll.1)-(ll.2)
W2<- ((1/N)*sum((ll.t)^2))-((1/N)*sum(ll.t))^2
W<-sqrt(W2)
LR<-(L1-L2)-((K1-K2)/2)*log(N)
Z_statistic<-LR/(sqrt(N)*W)
Z_statistic
pnorm(Z_statistic)
Z_statistic<1.96
}#Z_statistic
################Vuong_AIC&Vuong_BIC
{
N<-1258
p2<-27
p1<-52
ll.2<-ll.NB
ll.1<-ll.ZIPoisson
dli<- (ll.1)-(ll.2)
AIC_dlic<-dli+((p2-p1)/N)
BIC_dlic<-dli+((p2-p1)*log(N)/(2*N) )
```

```
SD<-sd(dli)
Vuong_AIC <- (SD * sqrt(N))^-1 * sum(AIC_dlic)
Vuong_AIC
Vuong_BIC <- (SD * sqrt(N))^-1 * sum(BIC_dlic)
Vuong_BIC
}#Vuong_AIC&Vuong_BIC
##############################################################################Poisson
{
X<-read.csv("E:\\maghale1-98.4.8\\maghale1-data.csv")
X<-as.matrix(X)
X
beta<-read.csv("E:\\maghale1-98.4.8\\maghale1-cofficients.csv")
beta1<-na.omit(beta$Poisson)
beta1<-as.matrix(beta1)
beta1
number<-read.csv("E:\\maghale1-98.4.8\\number.csv")
Y<-as.matrix(number)
Y
ita<-X%*%beta1
ita
lambda<- drop(exp(ita))
lambda
y <- drop(Y)
ll.Poisson<- -lambda + y * log(lambda) - log(factorial(y))
total.ll.Poisson <- sum(ll.Poisson)
total.ll.Poisson
}#Poisson
###############################################################################EEGR
{
X<-read.csv("E:\\maghale1-98.4.8\\maghale1-data.csv")
X<-as.matrix(X)
beta<-read.csv("E:\\maghale1-98.4.8\\maghale1-cofficients.csv")
beta<-na.omit(beta$EEGR)
beta<-as.matrix(beta)
beta1<-beta[1:26,]
c<-beta[27,]
number<-read.csv("E:\\maghale1-98.4.8\\number.csv")
Y<-as.matrix(number)
Y
ita<-X%*%beta1
ita
lambda<- 1/(1+drop(exp(-ita)))
lambda
y <- drop(Y)
ll.EEGR<-log((1-lambda**(y+1))**c-(1-lambda**(y))**c)
total.ll.EEGR <- sum(ll.EEGR)
total.ll.EEGR
}#EEGR
################P&EEGR
```

```
{
N<-1258
K1<-26
K2<-27
L1<-total.ll.Poisson
L2<-total.ll.EEGR
ll.1<-ll.Poisson
ll.2<-ll.EEGR
ll.t<- (ll.1)-(ll.2)
W2<- ((1/N)*sum((ll.t)^2))-((1/N)*sum(ll.t))^2
W<-sqrt(W2)
LR<-(L1-L2)-((K1-K2)/2)*log(N)
Z_statistic<-LR/(sqrt(N)*W)
Z_statistic
pnorm(Z_statistic)
Z_statistic<1.96
}#Z_statistic
################Vuong_AIC&Vuong_BIC
{
N<-1258
p2<-26
p1<-27
ll.2<-ll.Poisson
ll.1<-ll.EEGR
dli<- (ll.1)-(ll.2)
AIC_dlic<-dli+((p2-p1)/N)
BIC_dlic<-dli+((p2-p1)*log(N)/(2*N) )
SD<-sd(dli)
Vuong_AIC <- (SD * sqrt(N))^-1 * sum(AIC_dlic)
Vuong_AIC
Vuong_BIC <- (SD * sqrt(N))^-1 * sum(BIC_dlic)
Vuong_BIC
}#Vuong_AIC&Vuong_BIC
####################################################################################Poisson
{
X<-read.csv("E:\\maghale1-98.4.8\\maghale1-data.csv")
X<-as.matrix(X)
X
beta<-read.csv("E:\\maghale1-98.4.8\\maghale1-cofficients.csv")
beta1<-na.omit(beta$Poisson)
beta1<-as.matrix(beta1)
beta1
number<-read.csv("E:\\maghale1-98.4.8\\number.csv")
Y<-as.matrix(number)
Y
ita<-X%*%beta1
```

```
ita
lambda<- drop(exp(ita))
lambda
y <- drop(Y)
ll.Poisson<- -lambda + y * log(lambda) - log(factorial(y))
total.ll.Poisson <- sum(ll.Poisson)
total.ll.Poisson
}#Poisson
#########################################################################################ZIGP
{
X<-read.csv("E:\\maghale1-98.4.8\\maghale1-data.csv")
X<-as.matrix(X)
X
beta<-read.csv("E:\\maghale1-98.4.8\\maghale1-cofficients.csv")
beta<-na.omit(beta$GP)
beta<-as.matrix(beta)
beta1<-beta[1:26,]
beta1
alpha<-beta[27,]
number<-read.csv("E:\\maghale1-98.4.8\\number.csv")
Y<-as.matrix(number)
Y
y <- drop(Y)
ita<-X%*%beta1
ita
lambda<-drop(exp(ita))
lambda
ll.GP<- y*(log(lambda)-log(1+alpha*lambda))+(y-1)*log(1+alpha*y)-log(factorial(y))-
(lambda*(1+alpha*y))/(1+alpha*lambda)
total.ll.GP <- sum(ll.GP)
total.ll.GP
}#GP
################P&GP
{
N<-1258
K1<-26
K2<-27
L1<-total.ll.Poisson
L2<-total.ll.GP
ll.1<-ll.Poisson
ll.2<-ll.GP
ll.t<- (ll.1)-(ll.2)
W2<- ((1/N)*sum((ll.t)^2))-((1/N)*sum(ll.t))^2
W<-sqrt(W2)
LR<-(L1-L2)-((K1-K2)/2)*log(N)
Z_statistic<-LR/(sqrt(N)*W)
Z_statistic
pnorm(Z_statistic)
Z_statistic<1.96
```

```
}#Z_statistic
################Vuong_AIC&Vuong_BIC
{
N<-1258
p2<-26
p1<-27
ll.2<-ll.Poisson
ll.1<-ll.GP
dli<- (ll.1)-(ll.2)
AIC_dlic<-dli+((p2-p1)/N)
BIC_dlic<-dli+((p2-p1)*log(N)/(2*N) )
SD<-sd(dli)
Vuong_AIC <- (SD * sqrt(N))^-1 * sum(AIC_dlic)
Vuong_AIC
Vuong_BIC <- (SD * sqrt(N))^-1 * sum(BIC_dlic)
Vuong_BIC
}#Vuong_AIC&Vuong_BIC
##################################################################################Poisson
{
X<-read.csv("E:\\maghale1-98.4.8\\maghale1-data.csv")
X<-as.matrix(X)
X
beta<-read.csv("E:\\maghale1-98.4.8\\maghale1-cofficients.csv")
beta1<-na.omit(beta$Poisson)
beta1<-as.matrix(beta1)
beta1
number<-read.csv("E:\\maghale1-98.4.8\\number.csv")
Y<-as.matrix(number)
Y
ita<-X%*%beta1
ita
lambda<- drop(exp(ita))
lambda
y <- drop(Y)
ll.Poisson<- -lambda + y * log(lambda) - log(factorial(y))
total.ll.Poisson <- sum(ll.Poisson)
total.ll.Poisson
}#Poisson
#######################################################################################NB
{
X<-read.csv("E:\\maghale1-98.4.8\\maghale1-data.csv")
X<-as.matrix(X)
X
class(X)
beta<-read.csv("E:\\maghale1-98.4.8\\maghale1-cofficients.csv")
beta<-na.omit(beta$NB)
beta<-as.matrix(beta)
beta1<-beta[1:26,]
beta1
```

```
number<-read.csv("E:\\maghale1-98.4.8\\number.csv")
Y<-as.matrix(number)
Y
ita<-X%*%beta1
ita
lambda<- drop(exp(ita))
lambda
y <- drop(Y)
alpha<-beta[27,]
m = 1/alpha
p = 1/(1+alpha*lambda)
ll.NB<-log(gamma(m + y)) - log(gamma(y + 1))- log(gamma(m)) + (m*log(p)) + y*log(1-p)
total.ll.NB <- sum(ll.NB)
total.ll.NB
}#NB
################P&NB
{
N<-1258
K1<-26
K2<-27
L1<-total.ll.Poisson
L2<-total.ll.NB
ll.1<-ll.Poisson
ll.2<-ll.NB
ll.t<- (ll.1)-(ll.2)
W2<- ((1/N)*sum((ll.t)^2))-((1/N)*sum(ll.t))^2
W<-sqrt(W2)
LR<-(L1-L2)-((K1-K2)/2)*log(N)
Z_statistic<-LR/(sqrt(N)*W)
Z_statistic
pnorm(Z_statistic)
Z_statistic<1.96
}#Z_statistic
################Vuong_AIC&Vuong_BIC
{
N<-1258
p2<-26
p1<-27
ll.2<-ll.Poisson
ll.1<-ll.NB
dli<- (ll.1)-(ll.2)
AIC_dlic<-dli+((p2-p1)/N)
BIC_dlic<-dli+((p2-p1)*log(N)/(2*N) )
SD<-sd(dli)
Vuong_AIC <- (SD * sqrt(N))^-1 * sum(AIC_dlic)
Vuong_AIC
```

```
Vuong_BIC <- (SD * sqrt(N))^-1 * sum(BIC_dlic)
Vuong_BIC
}#Vuong_AIC&Vuong_BIC
##############################################################################Poisson
{
X<-read.csv("E:\\maghale1-98.4.8\\maghale1-data.csv")
X<-as.matrix(X)
X
beta<-read.csv("E:\\maghale1-98.4.8\\maghale1-cofficients.csv")
beta1<-na.omit(beta$Poisson)
beta1<-as.matrix(beta1)
beta1
number<-read.csv("E:\\maghale1-98.4.8\\number.csv")
Y<-as.matrix(number)
Y
ita<-X%*%beta1
ita
lambda<- drop(exp(ita))
lambda
y <- drop(Y)
ll.Poisson<- -lambda + y * log(lambda) - log(factorial(y))
total.ll.Poisson <- sum(ll.Poisson)
total.ll.Poisson
}#Poisson
#############################################################################ZIEEGR
{
X<-read.csv("E:\\maghale1-98.4.8\\maghale1-data.csv")
X<-as.matrix(X)
number<-read.csv("E:\\maghale1-98.4.8\\number.csv")
Y<-as.matrix(number)
Y
y <- drop(Y)
beta<-read.csv("E:\\maghale1-98.4.8\\maghale1-cofficients.csv")
ZIEEGR1<-na.omit(beta$ZIEEGR1)
ZIEEGR1<-as.matrix(ZIEEGR1)
beta1<-ZIEEGR1[1:26,]
c<-ZIEEGR1[27,]
c <- drop(c)
ita1<-X%*%beta1
ita1
lambda<-1/(1+drop(exp(-ita1)))
lambda
Z <- X
ZIEEGR2<-na.omit(beta$ZIEEGR2)
ZIEEGR2<-as.matrix(ZIEEGR2)
gamma1 <- ZIEEGR2
ita2<-Z%*%gamma1
ita2
P.loit <- 1/(1+drop(exp(-ita2)))
```

```
ll.count <- log(1-P.loit) + log((1-lambda^(y+1))^c-(1-lambda^(y))^c)
ll.zero <-log(P.loit + (1-P.loit)*(1-lambda)**c)
ll.ZIEEGR <- ifelse(y==0,ll.zero,ll.count)
total.ll.ZIEEGR<- sum(ll.ZIEEGR)
total.ll.ZIEEGR
}#ZIEEGR
###############P&ZIEEGR
{
N<-1258
K1<-26
K2<-53
L1<-total.ll.Poisson
L2<-total.ll.ZIEEGR
ll.1<-ll.Poisson
ll.2<-ll.ZIEEGR
ll.t<- (ll.1)-(ll.2)
W2<- ((1/N)*sum((ll.t)^2))-((1/N)*sum(ll.t))^2
W<-sqrt(W2)
LR<-(L1-L2)-((K1-K2)/2)*log(N)
Z_statistic<-LR/(sqrt(N)*W)
Z_statistic
pnorm(Z_statistic)
Z_statistic<1.96
}#Z_statistic
###############Vuong_AIC&Vuong_BIC
{
N<-1258
p2<-26
p1<-53
ll.2<-ll.Poisson
ll.1<-ll.ZIEEGR
dli<- (ll.1)-(ll.2)
AIC_dlic<-dli+((p2-p1)/N)
BIC_dlic<-dli+((p2-p1)*log(N)/(2*N) )
SD<-sd(dli)
Vuong_AIC <- (SD * sqrt(N))^-1 * sum(AIC_dlic)
Vuong_AIC
Vuong_BIC <- (SD * sqrt(N))^-1 * sum(BIC_dlic)
Vuong_BIC
}#Vuong_AIC&Vuong_BIC
##################################################################################Poisson
{
X<-read.csv("E:\\maghale1-98.4.8\\maghale1-data.csv")
X<-as.matrix(X)
X
beta<-read.csv("E:\\maghale1-98.4.8\\maghale1-cofficients.csv")
beta1<-na.omit(beta$Poisson)
beta1<-as.matrix(beta1)
```

```
beta1
number<-read.csv("E:\\maghale1-98.4.8\\number.csv")
Y<-as.matrix(number)
Y
ita<-X%*%beta1
ita
lambda<- drop(exp(ita))
lambda
y <- drop(Y)
ll.Poisson<- -lambda + y * log(lambda) - log(factorial(y))
total.ll.Poisson <- sum(ll.Poisson)
total.ll.Poisson
}#Poisson
###########################################################################################ZIGP
{
X<-read.csv("E:\\maghale1-98.4.8\\maghale1-data.csv")
X<-as.matrix(X)
X
number<-read.csv("E:\\maghale1-98.4.8\\number.csv")
Y<-as.matrix(number)
Y
y <- drop(Y)
beta<-read.csv("E:\\maghale1-98.4.8\\maghale1-cofficients.csv")
ZIGP1<-na.omit(beta$ZIGP1)
ZIGP1<-as.matrix(ZIGP1)
beta1<-ZIGP1[1:26,]
alpha<-ZIGP1[27,]
ita1<-X%*%beta1
ita1
lambda<- drop(exp(ita1))
lambda
Z <- X
beta<-read.csv("E:\\maghale1-98.4.8\\maghale1-cofficients.csv")
ZIGP2<-na.omit(beta$ZIGP2)
ZIGP2<-as.matrix(ZIGP2)
gamma1 <- ZIGP2
ita2<-Z%*%gamma1
ita2
P.loit <- 1/(1+drop(exp(-ita2)))
ll.count <- log(1-P.loit) +y*(log(lambda)-log(1+alpha*lambda))+(y-1)*log(1+alpha*y)-log(factorial(y))-
(lambda*(1+ alpha*y))/(1+alpha*lambda)
ll.zero <-log(P.loit + (1-P.loit)*exp(-lambda/(1+alpha*lambda)))
ll.ZIGP <- ifelse(y==0,ll.zero,ll.count)
total.ll.ZIGP<- sum(ll.ZIGP)
total.ll.ZIGP
}#ZIGP
```

```
################P&ZIGP
{
N<-1258
K1<-26
K2<-53
L1<-total.ll.Poisson
L2<-total.ll.ZIGP
ll.1<-ll.Poisson
ll.2<-ll.ZIGP
ll.t<- (ll.1)-(ll.2)
W2<- ((1/N)*sum((ll.t)^2))-((1/N)*sum(ll.t))^2
W<-sqrt(W2)
LR<-(L1-L2)-((K1-K2)/2)*log(N)
Z_statistic<-LR/(sqrt(N)*W)
Z_statistic
pnorm(Z_statistic)
Z_statistic<1.96
}#Z_statistic
################Vuong_AIC&Vuong_BIC
{
N<-1258
p2<-26
p1<-53
ll.2<-ll.Poisson
ll.1<-ll.ZIGP
dli<- (ll.1)-(ll.2)
AIC_dlic<-dli+((p2-p1)/N)
BIC_dlic<-dli+((p2-p1)*log(N)/(2*N) )
SD<-sd(dli)
Vuong_AIC <- (SD * sqrt(N))^-1 * sum(AIC_dlic)
Vuong_AIC
Vuong_BIC <- (SD * sqrt(N))^-1 * sum(BIC_dlic)
Vuong_BIC
}#Vuong_AIC&Vuong_BIC
####################################################################################Poisson
{
X<-read.csv("E:\\maghale1-98.4.8\\maghale1-data.csv")
X<-as.matrix(X)
X
beta<-read.csv("E:\\maghale1-98.4.8\\maghale1-cofficients.csv")
beta1<-na.omit(beta$Poisson)
beta1<-as.matrix(beta1)
beta1
number<-read.csv("E:\\maghale1-98.4.8\\number.csv")
Y<-as.matrix(number)
Y
ita<-X%*%beta1
ita
lambda<- drop(exp(ita))
```

```
lambda
y <- drop(Y)
ll.Poisson<- -lambda + y * log(lambda) - log(factorial(y))
total.ll.Poisson <- sum(ll.Poisson)
total.ll.Poisson
}#Poisson
########################################################################################ZINB
{
X<-read.csv("E:\\maghale1-98.4.8\\maghale1-data.csv")
X<-as.matrix(X)
X
number<-read.csv("E:\\maghale1-98.4.8\\number.csv")
Y<-as.matrix(number)
Y
y <- drop(Y)
beta<-read.csv("E:\\maghale1-98.4.8\\maghale1-cofficients.csv")
ZINB1<-na.omit(beta$ZINB1)
ZINB1<-as.matrix(ZINB1)
beta1<-ZINB1[1:26,]
alpha<-ZINB1[27,]
ita1<-X%*%beta1
ita1
lambda<- drop(exp(ita1))
lambda
Z <- X
beta<-read.csv("E:\\maghale1-98.4.8\\maghale1-cofficients.csv")
ZINB2<-na.omit(beta$ZINB2)
ZINB2<-as.matrix(ZINB2)
gamma1 <- ZINB2
ita2<-Z%*%gamma1
ita2
m = 1/alpha
p = 1/(1+alpha*lambda)
P.loit <- 1/(1+drop(exp(-ita2)))
ll.count <- log(1-P.loit) + log(gamma(m + y)) - log(gamma(y + 1))- log(gamma(m)) + (m*log(p)) + (y*log(1-
p))
ll.zero <-log(P.loit + (1-P.loit)*(p**m))
ll.ZINB <- ifelse(y==0,ll.zero,ll.count)
total.ll.ZINB<-sum(ll.ZINB)
total.ll.ZINB
}#ZINB
################P&ZINB
{
N<-1258
K1<-26
K2<-53
L1<-total.ll.Poisson
L2<-total.ll.ZINB
ll.1<-ll.Poisson
```

```
ll.2<-ll.ZINB
ll.t<- (ll.1)-(ll.2)
W2<- ((1/N)*sum((ll.t)^2))-((1/N)*sum(ll.t))^2
W<-sqrt(W2)
LR<-(L1-L2)-((K1-K2)/2)*log(N)
Z_statistic<-LR/(sqrt(N)*W)
Z_statistic
pnorm(Z_statistic)
Z_statistic<1.96
}#Z_statistic
################Vuong_AIC&Vuong_BIC
{
N<-1258
p2<-26
p1<-53
ll.2<-ll.Poisson
ll.1<-ll.ZINB
dli<- (ll.1)-(ll.2)
AIC_dlic<-dli+((p2-p1)/N)
BIC_dlic<-dli+((p2-p1)*log(N)/(2*N) )
SD<-sd(dli)
Vuong_AIC <- (SD * sqrt(N))^-1 * sum(AIC_dlic)
Vuong_AIC
Vuong_BIC <- (SD * sqrt(N))^-1 * sum(BIC_dlic)
Vuong_BIC
}#Vuong_AIC&Vuong_BIC
#####################################################################################Poisson
{
X<-read.csv("E:\\maghale1-98.4.8\\maghale1-data.csv")
X<-as.matrix(X)
X
beta<-read.csv("E:\\maghale1-98.4.8\\maghale1-cofficients.csv")
beta1<-na.omit(beta$Poisson)
beta1<-as.matrix(beta1)
beta1
number<-read.csv("E:\\maghale1-98.4.8\\number.csv")
Y<-as.matrix(number)
Y
ita<-X%*%beta1
ita
lambda<- drop(exp(ita))
lambda
y <- drop(Y)
ll.Poisson<- -lambda + y * log(lambda) - log(factorial(y))
total.ll.Poisson <- sum(ll.Poisson)
total.ll.Poisson
}#Poisson
#####################################################################################ZIPoisson
{
```

```
X<-read.csv("E:\\maghale1-98.4.8\\maghale1-data.csv")
X<-as.matrix(X)
X
number<-read.csv("E:\\maghale1-98.4.8\\number.csv")
Y<-as.matrix(number)
Y
y <- drop(Y)
beta<-read.csv("E:\\maghale1-98.4.8\\maghale1-cofficients.csv")
ZIP1<-na.omit(beta$ZIP1)
ZIP1<-as.matrix(ZIP1)
ita1<-X%*%ZIP1
ita1
lambda<- drop(exp(ita1))
lambda
Z <- X
beta<-read.csv("E:\\maghale1-98.4.8\\maghale1-cofficients.csv")
ZIP2<-na.omit(beta$ZIP2)
ZIP2<-as.matrix(ZIP2)
gamma1 <- ZIP2
ita2<-Z%*%gamma1
ita2
P.loit <- 1/(1+drop(exp(-ita2)))
ll.count <- log((1-P.loit))-lambda +(y*log(lambda))-(log(factorial(y)))
ll.zero <-log(P.loit + (1-P.loit) * exp(-lambda))
ll.ZIPoisson <- ifelse(y==0,ll.zero,ll.count)
total.ll.ZIPoisson <- sum(ll.ZIPoisson)
total.ll.ZIPoisson
}#ZIPoisson
################P&ZIP
{
N<-1258
K1<-26
K2<-52
L1<-total.ll.Poisson
L2<-total.ll.ZIPoisson
ll.1<-ll.Poisson
ll.2<-ll.ZIPoisson
ll.t<- (ll.1)-(ll.2)
W2<- ((1/N)*sum((ll.t)^2))-((1/N)*sum(ll.t))^2
W<-sqrt(W2)
LR<-(L1-L2)-((K1-K2)/2)*log(N)
Z_statistic<-LR/(sqrt(N)*W)
Z_statistic
pnorm(Z_statistic)
Z_statistic<1.96
}#Z_statistic
################Vuong_AIC&Vuong_BIC
{
```

```
N<-1258
p2<-26
p1<-52
ll.2<-ll.Poisson
ll.1<-ll.ZIPoisson
dli<- (ll.1)-(ll.2)
AIC_dlic<-dli+((p2-p1)/N)
BIC_dlic<-dli+((p2-p1)*log(N)/(2*N) )
SD<-sd(dli)
Vuong_AIC <- (SD * sqrt(N))^-1 * sum(AIC_dlic)
Vuong_AIC
Vuong_BIC <- (SD * sqrt(N))^-1 * sum(BIC_dlic)
Vuong_BIC
}#Vuong_AIC&Vuong_BIC
####################################################################################ZIGP
{
X<-read.csv("E:\\maghale1-98.4.8\\maghale1-data.csv")
X<-as.matrix(X)
X
number<-read.csv("E:\\maghale1-98.4.8\\number.csv")
Y<-as.matrix(number)
Y
y <- drop(Y)
beta<-read.csv("E:\\maghale1-98.4.8\\maghale1-cofficients.csv")
ZIGP1<-na.omit(beta$ZIGP1)
ZIGP1<-as.matrix(ZIGP1)
beta1<-ZIGP1[1:26,]
alpha<-ZIGP1[27,]
ita1<-X%*%beta1
ita1
lambda<- drop(exp(ita1))
lambda
Z <- X
beta<-read.csv("E:\\maghale1-98.4.8\\maghale1-cofficients.csv")
ZIGP2<-na.omit(beta$ZIGP2)
ZIGP2<-as.matrix(ZIGP2)
gamma1 <- ZIGP2
ita2<-Z%*%gamma1
ita2
P.loit <- 1/(1+drop(exp(-ita2)))
ll.count <- log(1-P.loit) +y*(log(lambda)-log(1+alpha*lambda))+(y-1)*log(1+alpha*y)-log(factorial(y))-
(lambda*(1+ alpha*y))/(1+alpha*lambda)
ll.zero <-log(P.loit + (1-P.loit)*exp(-lambda/(1+alpha*lambda)))
ll.ZIGP <- ifelse(y==0,ll.zero,ll.count)
total.ll.ZIGP<- sum(ll.ZIGP)
total.ll.ZIGP
}#ZIGP
####################################################################################ZIEEGR
{
```

```
X<-read.csv("E:\\maghale1-98.4.8\\maghale1-data.csv")
X<-as.matrix(X)
number<-read.csv("E:\\maghale1-98.4.8\\number.csv")
Y<-as.matrix(number)
Y
y <- drop(Y)
beta<-read.csv("E:\\maghale1-98.4.8\\maghale1-cofficients.csv")
ZIEEGR1<-na.omit(beta$ZIEEGR1)
ZIEEGR1<-as.matrix(ZIEEGR1)
beta1<-ZIEEGR1[1:26,]
c<-ZIEEGR1[27,]
c <- drop(c)
ita1<-X%*%beta1
ita1
lambda<-1/(1+drop(exp(-ita1)))
lambda
Z <- X
ZIEEGR2<-na.omit(beta$ZIEEGR2)
ZIEEGR2<-as.matrix(ZIEEGR2)
gamma1 <- ZIEEGR2
ita2<-Z%*%gamma1
ita2
P.loit <- 1/(1+drop(exp(-ita2)))
ll.count <- log(1-P.loit) + log((1-lambda^(y+1))^c-(1-lambda^(y))^c)
ll.zero <-log(P.loit + (1-P.loit)*(1-lambda)**c)
ll.ZIEEGR <- ifelse(y==0,ll.zero,ll.count)
total.ll.ZIEEGR<- sum(ll.ZIEEGR)
total.ll.ZIEEGR
}#ZIEEGR
################ZIGP&ZIEEGR
{
N<-1258
K1<-53
K2<-53
L1<-total.ll.ZIGP
L2<-total.ll.ZIEEGR
ll.1<-ll.ZIGP
ll.2<-ll.ZIEEGR
ll.t<- (ll.1)-(ll.2)
W2<- ((1/N)*sum((ll.t)^2))-((1/N)*sum(ll.t))^2
W<-sqrt(W2)
LR<-(L1-L2)-((K1-K2)/2)*log(N)
Z_statistic<-LR/(sqrt(N)*W)
Z_statistic
pnorm(Z_statistic)
Z_statistic<1.96
}#Z_statistic
################Vuong_AIC&Vuong_BIC
```

```
{
N<-1258
p2<-53
p1<-53
ll.2<-ll.ZIGP
ll.1<-ll.ZIEEGR
dli<- (ll.1)-(ll.2)
AIC_dlic<-dli+((p2-p1)/N)
BIC_dlic<-dli+((p2-p1)*log(N)/(2*N) )
SD<-sd(dli)
Vuong_AIC <- (SD * sqrt(N))^-1 * sum(AIC_dlic)
Vuong_AIC
Vuong_BIC <- (SD * sqrt(N))^-1 * sum(BIC_dlic)
Vuong_BIC
}#Vuong_AIC&Vuong_BIC
#####################################################################################ZINB
{
X<-read.csv("E:\\maghale1-98.4.8\\maghale1-data.csv")
X<-as.matrix(X)
X
number<-read.csv("E:\\maghale1-98.4.8\\number.csv")
Y<-as.matrix(number)
Y
y <- drop(Y)
beta<-read.csv("E:\\maghale1-98.4.8\\maghale1-cofficients.csv")
ZINB1<-na.omit(beta$ZINB1)
ZINB1<-as.matrix(ZINB1)
beta1<-ZINB1[1:26,]
alpha<-ZINB1[27,]
ita1<-X%*%beta1
ita1
lambda<- drop(exp(ita1))
lambda
Z <- X
beta<-read.csv("E:\\maghale1-98.4.8\\maghale1-cofficients.csv")
ZINB2<-na.omit(beta$ZINB2)
ZINB2<-as.matrix(ZINB2)
gamma1 <- ZINB2
ita2<-Z%*%gamma1
ita2
m = 1/alpha
p = 1/(1+alpha*lambda)
P.loit <- 1/(1+drop(exp(-ita2)))
ll.count <- log(1-P.loit) + log(gamma(m + y)) - log(gamma(y + 1))- log(gamma(m)) + (m*log(p)) + (y*log(1-
p))
ll.zero <-log(P.loit + (1-P.loit)*(p**m))
ll.ZINB <- ifelse(y==0,ll.zero,ll.count)
total.ll.ZINB<-sum(ll.ZINB)
total.ll.ZINB
```

```
}#ZINB
####################################################################################ZIEEGR
{
X<-read.csv("E:\\maghale1-98.4.8\\maghale1-data.csv")
X<-as.matrix(X)
number<-read.csv("E:\\maghale1-98.4.8\\number.csv")
Y<-as.matrix(number)
Y
y <- drop(Y)
beta<-read.csv("E:\\maghale1-98.4.8\\maghale1-cofficients.csv")
ZIEEGR1<-na.omit(beta$ZIEEGR1)
ZIEEGR1<-as.matrix(ZIEEGR1)
beta1<-ZIEEGR1[1:26,]
c<-ZIEEGR1[27,]
c <- drop(c)
ita1<-X%*%beta1
ita1
lambda<-1/(1+drop(exp(-ita1)))
lambda
Z <- X
ZIEEGR2<-na.omit(beta$ZIEEGR2)
ZIEEGR2<-as.matrix(ZIEEGR2)
gamma1 <- ZIEEGR2
ita2<-Z%*%gamma1
ita2
P.loit <- 1/(1+drop(exp(-ita2)))
ll.count <- log(1-P.loit) + log((1-lambda^(y+1))^c-(1-lambda^(y))^c)
ll.zero <-log(P.loit + (1-P.loit)*(1-lambda)**c)
ll.ZIEEGR <- ifelse(y==0,ll.zero,ll.count)
total.ll.ZIEEGR<- sum(ll.ZIEEGR)
total.ll.ZIEEGR
}#ZIEEGR
################ZINB&ZIEEGR
{
N<-1258
K1<-53
K2<-53
L1<-total.ll.ZINB
L2<-total.ll.ZIEEGR
ll.1<-ll.ZINB
ll.2<-ll.ZIEEGR
ll.t<- (ll.1)-(ll.2)
W2<- ((1/N)*sum((ll.t)^2))-((1/N)*sum(ll.t))^2
W<-sqrt(W2)
LR<-(L1-L2)-((K1-K2)/2)*log(N)
Z_statistic<-LR/(sqrt(N)*W)
Z_statistic
pnorm(Z_statistic)
```

```
Z_statistic<1.96
}#Z_statistic
################Vuong_AIC&Vuong_BIC
{
N<-1258
p2<-53
p1<-53
ll.2<-ll.ZINB
ll.1<-ll.ZIEEGR
dli<- (ll.1)-(ll.2)
AIC_dlic<-dli+((p2-p1)/N)
BIC_dlic<-dli+((p2-p1)*log(N)/(2*N) )
SD<-sd(dli)
Vuong_AIC <- (SD * sqrt(N))^-1 * sum(AIC_dlic)
Vuong_AIC
Vuong_BIC <- (SD * sqrt(N))^-1 * sum(BIC_dlic)
Vuong_BIC
}#Vuong_AIC&Vuong_BIC
##########################################################################################ZINB
{
X<-read.csv("E:\\maghale1-98.4.8\\maghale1-data.csv")
X<-as.matrix(X)
X
number<-read.csv("E:\\maghale1-98.4.8\\number.csv")
Y<-as.matrix(number)
Y
y <- drop(Y)
beta<-read.csv("E:\\maghale1-98.4.8\\maghale1-cofficients.csv")
ZINB1<-na.omit(beta$ZINB1)
ZINB1<-as.matrix(ZINB1)
beta1<-ZINB1[1:26,]
alpha<-ZINB1[27,]
ita1<-X%*%beta1
ita1
lambda<- drop(exp(ita1))
lambda
Z <- X
beta<-read.csv("E:\\maghale1-98.4.8\\maghale1-cofficients.csv")
ZINB2<-na.omit(beta$ZINB2)
ZINB2<-as.matrix(ZINB2)
gamma1 <- ZINB2
ita2<-Z%*%gamma1
ita2
m = 1/alpha
p = 1/(1+alpha*lambda)
P.loit <- 1/(1+drop(exp(-ita2)))
ll.count <- log(1-P.loit) + log(gamma(m + y)) - log(gamma(y + 1))- log(gamma(m)) + (m*log(p)) + (y*log(1-
p))
ll.zero <-log(P.loit + (1-P.loit)*(p**m))
```

```
ll.ZINB <- ifelse(y==0,ll.zero,ll.count)
total.ll.ZINB<-sum(ll.ZINB)
total.ll.ZINB
}#ZINB
##########################################################################################ZIGP
{
X<-read.csv("E:\\maghale1-98.4.8\\maghale1-data.csv")
X<-as.matrix(X)
X
number<-read.csv("E:\\maghale1-98.4.8\\number.csv")
Y<-as.matrix(number)
Y
y <- drop(Y)
beta<-read.csv("E:\\maghale1-98.4.8\\maghale1-cofficients.csv")
ZIGP1<-na.omit(beta$ZIGP1)
ZIGP1<-as.matrix(ZIGP1)
beta1<-ZIGP1[1:26,]
alpha<-ZIGP1[27,]
ita1<-X%*%beta1
ita1
lambda<- drop(exp(ita1))
lambda
Z <- X
beta<-read.csv("E:\\maghale1-98.4.8\\maghale1-cofficients.csv")
ZIGP2<-na.omit(beta$ZIGP2)
ZIGP2<-as.matrix(ZIGP2)
gamma1 <- ZIGP2
ita2<-Z%*%gamma1
ita2
P.loit <- 1/(1+drop(exp(-ita2)))
ll.count <- log(1-P.loit) +y*(log(lambda)-log(1+alpha*lambda))+(y-1)*log(1+alpha*y)-log(factorial(y))-
(lambda*(1+ alpha*y))/(1+alpha*lambda)
ll.zero <-log(P.loit + (1-P.loit)*exp(-lambda/(1+alpha*lambda)))
ll.ZIGP <- ifelse(y==0,ll.zero,ll.count)
total.ll.ZIGP<- sum(ll.ZIGP)
total.ll.ZIGP
}#ZIGP
################ZINB&ZIGP
{
N<-1258
K1<-53
K2<-53
L1<-total.ll.ZINB
L2<-total.ll.ZIGP
ll.1<-ll.ZINB
ll.2<-ll.ZIGP
ll.t<- (ll.1)-(ll.2)
W2<- ((1/N)*sum((ll.t)^2))-((1/N)*sum(ll.t))^2
```

```
W<-sqrt(W2)
LR<-(L1-L2)-((K1-K2)/2)*log(N)
Z_statistic<-LR/(sqrt(N)*W)
Z_statistic
pnorm(Z_statistic)
Z_statistic<1.96
}#Z_statistic
################Vuong_AIC&Vuong_BIC
{
N<-1258
p2<-53
p1<-53
ll.2<-ll.ZINB
ll.1<-ll.ZIGP
dli<- (ll.1)-(ll.2)
AIC_dlic<-dli+((p2-p1)/N)
BIC_dlic<-dli+((p2-p1)*log(N)/(2*N) )
SD<-sd(dli)
Vuong_AIC <- (SD * sqrt(N))^-1 * sum(AIC_dlic)
Vuong_AIC
Vuong_BIC <- (SD * sqrt(N))^-1 * sum(BIC_dlic)
Vuong_BIC
}#Vuong_AIC&Vuong_BIC
##################################################################################ZIPoisson
{
X<-read.csv("E:\\maghale1-98.4.8\\maghale1-data.csv")
X<-as.matrix(X)
X
number<-read.csv("E:\\maghale1-98.4.8\\number.csv")
Y<-as.matrix(number)
Y
y <- drop(Y)
beta<-read.csv("E:\\maghale1-98.4.8\\maghale1-cofficients.csv")
ZIP1<-na.omit(beta$ZIP1)
ZIP1<-as.matrix(ZIP1)
ita1<-X%*%ZIP1
ita1
lambda<- drop(exp(ita1))
lambda
Z <- X
number<-read.csv("E:\\maghale1-98.4.8\\number.csv")
ZIP2<-na.omit(beta$ZIP2)
ZIP2<-as.matrix(ZIP2)
gamma1 <- ZIP2
ita2<-Z%*%gamma1
ita2
P.loit <- 1/(1+drop(exp(-ita2)))
ll.count <- log((1-P.loit))-lambda +(y*log(lambda))-(log(factorial(y)))
ll.zero <-log(P.loit + (1-P.loit) * exp(-lambda))
```

```
ll.ZIP <- ifelse(y==0,ll.zero,ll.count)
total.ll.ZIP<- sum(ll.ZIP)
total.ll.ZIP
}#ZIP
####################################################################################ZIEEGR
{
X<-read.csv("E:\\maghale1-98.4.8\\maghale1-data.csv")
X<-as.matrix(X)
number<-read.csv("E:\\maghale1-98.4.8\\number.csv")
Y<-as.matrix(number)
Y
y <- drop(Y)
beta<-read.csv("E:\\maghale1-98.4.8\\maghale1-cofficients.csv")
ZIEEGR1<-na.omit(beta$ZIEEGR1)
ZIEEGR1<-as.matrix(ZIEEGR1)
beta1<-ZIEEGR1[1:26,]
c<-ZIEEGR1[27,]
c <- drop(c)
ita1<-X%*%beta1
ita1
lambda<-1/(1+drop(exp(-ita1)))
lambda
Z <- X
ZIEEGR2<-na.omit(beta$ZIEEGR2)
ZIEEGR2<-as.matrix(ZIEEGR2)
gamma1 <- ZIEEGR2
ita2<-Z%*%gamma1
ita2
P.loit <- 1/(1+drop(exp(-ita2)))
ll.count <- log(1-P.loit) + log((1-lambda^(y+1))^c-(1-lambda^(y))^c)
ll.zero <-log(P.loit + (1-P.loit)*(1-lambda)**c)
ll.ZIEEGR <- ifelse(y==0,ll.zero,ll.count)
total.ll.ZIEEGR<- sum(ll.ZIEEGR)
total.ll.ZIEEGR
}#ZIEEGR
################ZIP&ZIEEGR
{
N<-1258
K1<-52
K2<-53
L1<-total.ll.ZIP
L2<-total.ll.ZIEEGR
ll.1<-ll.ZIP
ll.2<-ll.ZIEEGR
ll.t<- (ll.1)-(ll.2)
W2<- ((1/N)*sum((ll.t)^2))-((1/N)*sum(ll.t))^2
W<-sqrt(W2)
LR<-(L1-L2)-((K1-K2)/2)*log(N)
```

```
Z_statistic<-LR/(sqrt(N)*W)
Z_statistic
pnorm(Z_statistic)
Z_statistic<1.96
}#Z_statistic
################Vuong_AIC&Vuong_BIC
{
N<-1258
p2<-52
p1<-53
ll.2<-ll.ZIP
ll.1<-ll.ZIEEGR
dli<- (ll.1)-(ll.2)
AIC_dlic<-dli+((p2-p1)/N)
BIC_dlic<-dli+((p2-p1)*log(N)/(2*N) )
SD<-sd(dli)
Vuong_AIC <- (SD * sqrt(N))^-1 * sum(AIC_dlic)
Vuong_AIC
Vuong_BIC <- (SD * sqrt(N))^-1 * sum(BIC_dlic)
Vuong_BIC
}#Vuong_AIC&Vuong_BIC
####################################################################################ZIPoisson
{
X<-read.csv("E:\\maghale1-98.4.8\\maghale1-data.csv")
X<-as.matrix(X)
X
number<-read.csv("E:\\maghale1-98.4.8\\number.csv")
Y<-as.matrix(number)
Y
y <- drop(Y)
beta<-read.csv("E:\\maghale1-98.4.8\\maghale1-cofficients.csv")
ZIP1<-na.omit(beta$ZIP1)
ZIP1<-as.matrix(ZIP1)
ita1<-X%*%ZIP1
ita1
lambda<- drop(exp(ita1))
lambda
Z <- X
number<-read.csv("E:\\maghale1-98.4.8\\number.csv")
ZIP2<-na.omit(beta$ZIP2)
ZIP2<-as.matrix(ZIP2)
gamma1 <- ZIP2
ita2<-Z%*%gamma1
ita2
P.loit <- 1/(1+drop(exp(-ita2)))
ll.count <- log((1-P.loit))-lambda +(y*log(lambda))-(log(factorial(y)))
ll.zero <-log(P.loit + (1-P.loit) * exp(-lambda))
```

```
ll.ZIP <- ifelse(y==0,ll.zero,ll.count)
total.ll.ZIP<- sum(ll.ZIP)
total.ll.ZIP
}#ZIP
##########################################################################################ZIGP
{
X<-read.csv("E:\\maghale1-98.4.8\\maghale1-data.csv")
X<-as.matrix(X)
X
number<-read.csv("E:\\maghale1-98.4.8\\number.csv")
Y<-as.matrix(number)
Y
y <- drop(Y)
beta<-read.csv("E:\\maghale1-98.4.8\\maghale1-cofficients.csv")
ZIGP1<-na.omit(beta$ZIGP1)
ZIGP1<-as.matrix(ZIGP1)
beta1<-ZIGP1[1:26,]
alpha<-ZIGP1[27,]
ita1<-X%*%beta1
ita1
lambda<- drop(exp(ita1))
lambda
Z <- X
beta<-read.csv("E:\\maghale1-98.4.8\\maghale1-cofficients.csv")
ZIGP2<-na.omit(beta$ZIGP2)
ZIGP2<-as.matrix(ZIGP2)
gamma1 <- ZIGP2
ita2<-Z%*%gamma1
ita2
P.loit <- 1/(1+drop(exp(-ita2)))
ll.count <- log(1-P.loit) +y*(log(lambda)-log(1+alpha*lambda))+(y-1)*log(1+alpha*y)-log(factorial(y))-
(lambda*(1+ alpha*y))/(1+alpha*lambda)
ll.zero <-log(P.loit + (1-P.loit)*exp(-lambda/(1+alpha*lambda)))
ll.ZIGP <- ifelse(y==0,ll.zero,ll.count)
total.ll.ZIGP<- sum(ll.ZIGP)
total.ll.ZIGP
}#ZIGP
################ZIP&ZIGP
{
N<-1258
K1<-52
K2<-53
L1<-total.ll.ZIP
L2<-total.ll.ZIGP
ll.1<-ll.ZIP
ll.2<-ll.ZIGP
ll.t<- (ll.1)-(ll.2)
W2<- ((1/N)*sum((ll.t)^2))-((1/N)*sum(ll.t))^2
```

```
W<-sqrt(W2)
LR<-(L1-L2)-((K1-K2)/2)*log(N)
Z_statistic<-LR/(sqrt(N)*W)
Z_statistic
pnorm(Z_statistic)
Z_statistic<1.96
}#Z_statistic
################Vuong_AIC&Vuong_BIC
{
N<-1258
p2<-52
p1<-53
ll.2<-ll.ZIP
ll.1<-ll.ZIGP
dli<- (ll.1)-(ll.2)
AIC_dlic<-dli+((p2-p1)/N)
BIC_dlic<-dli+((p2-p1)*log(N)/(2*N) )
SD<-sd(dli)
Vuong_AIC <- (SD * sqrt(N))^-1 * sum(AIC_dlic)
Vuong_AIC
Vuong_BIC <- (SD * sqrt(N))^-1 * sum(BIC_dlic)
Vuong_BIC
}#Vuong_AIC&Vuong_BIC
####################################################################################ZIPoisson
{
X<-read.csv("E:\\maghale1-98.4.8\\maghale1-data.csv")
X<-as.matrix(X)
X
number<-read.csv("E:\\maghale1-98.4.8\\number.csv")
Y<-as.matrix(number)
Y
y <- drop(Y)
beta<-read.csv("E:\\maghale1-98.4.8\\maghale1-cofficients.csv")
ZIP1<-na.omit(beta$ZIP1)
ZIP1<-as.matrix(ZIP1)
ita1<-X%*%ZIP1
ita1
lambda<- drop(exp(ita1))
lambda
Z <- X
number<-read.csv("E:\\maghale1-98.4.8\\number.csv")
ZIP2<-na.omit(beta$ZIP2)
ZIP2<-as.matrix(ZIP2)
gamma1 <- ZIP2
ita2<-Z%*%gamma1
ita2
P.loit <- 1/(1+drop(exp(-ita2)))
ll.count <- log((1-P.loit))-lambda +(y*log(lambda))-(log(factorial(y)))
ll.zero <-log(P.loit + (1-P.loit) * exp(-lambda))
```

```
ll.ZIP <- ifelse(y==0,ll.zero,ll.count)
total.ll.ZIP<- sum(ll.ZIP)
total.ll.ZIP
}#ZIP
####################################################################################ZINB
{
X<-read.csv("E:\\maghale1-98.4.8\\maghale1-data.csv")
X<-as.matrix(X)
X
number<-read.csv("E:\\maghale1-98.4.8\\number.csv")
Y<-as.matrix(number)
Y
y <- drop(Y)
beta<-read.csv("E:\\maghale1-98.4.8\\maghale1-cofficients.csv")
ZINB1<-na.omit(beta$ZINB1)
ZINB1<-as.matrix(ZINB1)
beta1<-ZINB1[1:26,]
alpha<-ZINB1[27,]
ita1<-X%*%beta1
ita1
lambda<- drop(exp(ita1))
lambda
Z <- X
beta<-read.csv("E:\\maghale1-98.4.8\\maghale1-cofficients.csv")
ZINB2<-na.omit(beta$ZINB2)
ZINB2<-as.matrix(ZINB2)
gamma1 <- ZINB2
ita2<-Z%*%gamma1
ita2
m = 1/alpha
p = 1/(1+alpha*lambda)
P.loit <- 1/(1+drop(exp(-ita2)))
ll.count <- log(1-P.loit) + log(gamma(m + y)) - log(gamma(y + 1))- log(gamma(m)) + (m*log(p)) + (y*log(1-
p))
ll.zero <-log(P.loit + (1-P.loit)*(p**m))
ll.ZINB <- ifelse(y==0,ll.zero,ll.count)
total.ll.ZINB<-sum(ll.ZINB)
total.ll.ZINB
}#ZINB
################ZIP&ZINB
{
N<-1258
K1<-52
K2<-53
L1<-total.ll.ZIP
L2<-total.ll.ZINB
ll.1<-ll.ZIP
```

```
ll.2<-ll.ZINB
ll.t<- (ll.1)-(ll.2)
W2<- ((1/N)*sum((ll.t)^2))-((1/N)*sum(ll.t))^2
W<-sqrt(W2)
LR<-(L1-L2)-((K1-K2)/2)*log(N)
Z_statistic<-LR/(sqrt(N)*W)
Z_statistic
pnorm(Z_statistic)
Z_statistic<1.96
}#Z_statistic
################Vuong_AIC&Vuong_BIC
{
N<-1258
p2<-52
p1<-53
ll.2<-ll.ZIP
ll.1<-ll.ZINB
dli<- (ll.1)-(ll.2)
AIC_dlic<-dli+((p2-p1)/N)
BIC_dlic<-dli+((p2-p1)*log(N)/(2*N) )
SD<-sd(dli)
Vuong_AIC <- (SD * sqrt(N))^-1 * sum(AIC_dlic)
Vuong_AIC
Vuong_BIC <- (SD * sqrt(N))^-1 * sum(BIC_dlic)
Vuong_BIC
}#Vuong_AIC&Vuong_BIC
```
