# Supplementary material for: Determining correlates of the average number of cigarette smoking among college students using count regression models
Source: Sci Rep. 2020 Jun 1;10:8874. doi: 10.1038/s41598-020-65813-4 (PMC7264191; doi:10.1038/s41598-020-65813-4)
Supplement: Supplementary file 2 — Supplementary File 1 [file 41598_2020_65813_MOESM2_ESM.docx]

Title page

**Determining correlates of the average number of cigarette smoking among college students using count regression models**

Sharareh Parami

Department of Biostatistics, School of Public Health, Hamadan University of Medical Sciences, Hamadan, Iran

Email: sh.parami@edu.umsha.ac.ir

Leili Tapak

Department of Biostatistics, School of Public Health, Hamadan University of Medical Sciences, Hamadan, Iran

Modeling of Noncommunicable Diseases Research center, Hamadan University of Medical Sciences, Hamadan, Iran

Email: [l.tapak@umsha.ac.ir](mailto:l.tapak@umsha.ac.ir)

Abbas Moghimbeigi

Department of Biostatistics, School of Public health, Alborz University of Medical Sciences, Alborz, Iran

Email: moghimbeigi@umsha.ac.ir

Jalal Poorolajal

Department of Epidemiology, School of Public Health, Hamadan University of Medical Sciences, Hamadan, Iran

Research Center for Health Sciences, Hamadan University of Medical Sciences, Hamadan, Iran

Email: [poorolajal@umsha.ac.ir](http://sph.umsha.ac.ir/uploads/dr.poorolajal.docx)

Ali Ghaleiha

Department of Psychiatry, School of Medicine, Hamadan University of Medical Sciences, Hamadan, Iran

Behavioral Disorders and Substance Abuse Research Center, Hamadan University of Medical Sciences, Hamadan, Iran

Email: [Ghaleiha@umsha.ac.ir](mailto:Ghaleiha@umsha.ac.ir)

Corresponding Author: Leili Tapak

Assistant Professor of Biostatistics, Department of Biostatistics, School of Public Health, Hamadan University of Medical Sciences, Hamadan, Iran

Email: [l.tapak@umsha.ac.ir](mailto:l.tapak@umsha.ac.ir)

Tel: 0098 918 110 3161

**Supplementary file 1**

**#########################SAS codes for model fitting####################**

**Code_** **EEGR**

**proc** **nlmixed** data=data tech=congra;

parameters b0=**0** b1=**0** b2=**0** b3=**0** bc1=**0** bc2=**0** b6=**0** b7=**0** b8=**0** b9=**0** b10=**0** b11=**0** b12=**0** b13=**0** b14=**0** b15=**0** b16=**0** b17=**0** b18=**0** b19=**0** c=**1**;

xc1=**0**; xc2=**0**;

bounds c>**0**;

if (city eq **1**) then xc1=**1**;

if (city eq **2**) then xc2=**1**;

lambda = **1**/(**1**+exp(-(b0 + b1*sex + b2*order + b3*mar + bc1*xc1 + bc2*xc2 + b6*con + b7*edu + b8*average + b9*interest + b10*optimistic + b11*boygirl + b12*breakdown + b13*hetero + b14*homo + b15*drug + b16*suicidetlt + b17*suicidealt + b18*social + b19*GHQ28gr)));

if number=**0** then

ll = log((**1**-lambda)**c);

else ll = log((**1**-lambda**( number+**1**))**c-(**1**-lambda**(number))**c);

model number ~ general(ll);

**run**;

**Code_** **GP**

**proc** **nlmixed** data=data;

parameters b0=**0** b1=**0** b2=**0** b3=**0** bc1=**0** bc2=**0** b6=**0** b7=**0** b8=**0** b9=**0** b10=**0** b11=**0** b12=**0** b13=**0** b14=**0** b15=**0** b16=**0** b17=**0** b18=**0** b19=**0** alpha = **1**;

xc1=**0**; xc2=**0**;

if (city eq **1**) then xc1=**1**;

if (city eq **2**) then xc2=**1**;

/* negative binomial with mean-dispersion */

lambda = exp(b0 + b1*sex + b2*order + b3*mar + bc1*xc1 + bc2*xc2 + b6*con + b7*edu + b8*average + b9*interest + b10*optimistic + b11*boygirl + b12*breakdown + b13*hetero + b14*homo + b15*drug + b16*suicidetlt + b17*suicidealt + b18*social + b19*GHQ28gr);

ll = number * (log(lambda) - log(**1**+alpha * lambda)) + (number-**1**) * log (**1**+alpha * number) - log (fact(number)) - lambda * (**1**+ alpha * number) / (**1**+alpha*lambda);

model number ~ general(ll);

**run**;

**Code_** **NB**

**proc** **nlmixed** data=data;

parameters b0=**0** b1=**0** b2=**0** b3=**0** bc1=**0** bc2=**0** b6=**0** b7=**0** b8=**0** b9=**0** b10=**0** b11=**0** b12=**0** b13=**0** b14=**0** b15=**0** b16=**0** b17=**0** b18=**0** b19=**0** alpha = **1**;

xc1=**0**; xc2=**0**;

if (city eq **1**) then xc1=**1**;

if (city eq **2**) then xc2=**1**;

/* negative binomial with mean-dispersion */

lambda = exp(b0 + b1*sex + b2*order + b3*mar + bc1*xc1 + bc2*xc2 + b6*con + b7*edu + b8*average+ b9*interest + b10*optimistic + b11*boygirl + b12*breakdown + b13*hetero + b14*homo + b15*drug + b16*suicidetlt + b17*suicidealt + b18*social + b19*GHQ28gr);

/* Build the ZIP log likelihood */

m = **1**/alpha;

p = **1**/(**1**+alpha*lambda);

ll = log(gamma(m + number)) - log(gamma(number + **1**)) - log(gamma(m)) + m*log(p) + number*log(**1**-p);

model number ~ general(ll);

**run**;

**Code_** **Poisson**

**proc** **nlmixed** data=data;

parameters b0=**0** b1=**0** b2=**0** b3=**0** bc1=**0** bc2=**0** b6=**0** b7=**0** b8=**0** b9=**0** b10=**0** b11=**0** b12=**0** b13=**0** b14=**0** b15=**0** b16=**0** b17=**0** b18=**0** b19=**0**;

xc1=**0**; xc2=**0**;

if (city eq **1**) then xc1=**1**;

if (city eq **2**) then xc2=**1**;

/* Poisson mean */

lambda = exp(b0 + b1*sex + b2*order + b3*mar + bc1*xc1 + bc2*xc2 + b6*con + b7*edu + b8*average+ b9*interest + b10*optimistic + b11*boygirl + b12*breakdown + b13*hetero + b14*homo + b15*drug + b16*suicidetlt + b17*suicidealt + b18*social + b19*GHQ28gr);

ll = - lambda + number *log(lambda) - lgamma(number + **1**);

model number ~ general(ll);

**run**;

**Code_** **ZIEEGR**

**proc** **nlmixed** data=data;

parameters b0=**0** b1=**0** b2=**0** b3=**0** bc1=**0** bc2=**0** b6=**0** b7=**0** b8=**0** b9=**0** b10=**0** b11=**0** b12=**0** b13=**0** b14=**0** b15=**0** b16=**0** b17=**0** b18=**0** b19=**0**

a0=**0** a1=**0** a2=**0** a3=**0** ac1=**0** ac2=**0** a6=**0** a7=**0** a8=**0** a9=**0** a10=**0** a11=**0** a12=**0** a13=**0** a14=**0** a15=**0** a16=**0** a17=**0** a18=**0** a19=**0** c=**1**;

xc1=**0**; xc2=**0**;

if (city eq **1**) then xc1=**1**;

if (city eq **2**) then xc2=**1**;

linpinfl = a0 + a1*sex + a2*order + a3*mar + ac1*xc1 + ac2*xc2 + a6*con + a7*edu + a8*average + a9*interest + a10*optimistic + a11*boygirl + a12*breakdown + a13*hetero + a14*homo + a15*drug + a16*suicidetlt + a17*suicidealt + a18*social + a19*GHQ28gr;

/* infprob = inflation probability for zeros */

/* = logistic transform of the linear predictor*/

infprob = **1**/(**1**+exp(-linpinfl));

lambda = **1**/(**1**+exp(-(b0 + b1*sex + b2*order + b3*mar + bc1*xc1 + bc2*xc2 + b6*con + b7*edu + b8* average + b9*interest + b10*optimistic + b11*boygirl + b12*breakdown + b13*hetero + b14*homo + b15*drug + b16*suicidetlt + b17*suicidealt + b18*social + b19*GHQ28gr)));

/* Build the ZIEEGR log likelihood */

if number=**0** then

ll = log(infprob + (**1**-infprob)*(**1**-lambda)**c);

else ll = log((**1**-infprob)) + log((**1**-lambda**(number+**1**))**c-(**1**-

lambda**(number))**c);

model number ~ general(ll);

**run**;

**Code_** **ZIGP**

**proc** **nlmixed** data=data;

parameters b0=**0** b1=**0** b2=**0** b3=**0** bc1=**0** bc2=**0** b6=**0** b7=**0** b8=**0** b9=**0** b10=**0** b11=**0** b12=**0** b13=**0** b14=**0** b15=**0** b16=**0** b17=**0** b18=**0** b19=**0**

a0=**0** a1=**0** a2=**0** a3=**0** ac1=**0** ac2=**0** a6=**0** a7=**0** a8=**0** a9=**0** a10=**0** a11=**0** a12=**0** a13=**0** a14=**0** a15=**0** a16=**0** a17=**0** a18=**0** a19=**0** alpha = **1**;

xc1=**0**; xc2=**0**;

if (city eq **1**) then xc1=**1**;

if (city eq **2**) then xc2=**1**;

/* linear predictor for the inflation probabilitY */

linpinfl = a0 + a1*sex + a2*order + a3*mar + ac1*xc1 + ac2*xc2 + a6*con + a7*edu + a8*average + a9*interest + a10*optimistic + a11*boygirl + a12*breakdown + a13*hetero + a14*homo + a15*drug + a16*suicidetlt + a17*suicidealt + a18*social + a19*GHQ28gr;

/* infprob = inflation probabilitY for zeros */

/* = logistic transform of the linear predictor*/

infprob = **1**/(**1**+exp(-linpinfl));

lambda = exp(b0 + b1*sex + b2*order + b3*mar + bc1*xc1 + bc2*xc2 + b6*con + b7*edu + b8*average + b9*interest + b10*optimistic + b11*boygirl + b12*breakdown + b13*hetero + b14*homo + b15*drug + b16*suicidetlt + b17*suicidealt + b18*social + b19*GHQ28gr);

if number=**0** then

ll = log(infprob + (**1**-infprob)*exp(-lambda/(**1**+alpha*lambda)));

else ll = log(**1**-infprob) + number * (log(lambda) - log(**1**+alpha * lambda)) + (number-**1**) * log (**1**+alpha * number) - log (fact(number))

- lambda * (**1**+ alpha * number) / (**1**+alpha*lambda);

model number ~ general(ll);

**run**;

**Code_** **ZINB**

**proc** **nlmixed** data=data;

parameters b0=**0** b1=**0** b2=**0** b3=**0** bc1=**0** bc2=**0** b6=**0** b7=**0** b8=**0** b9=**0** b10=**0** b11=**0** b12=**0** b13=**0** b14=**0** b15=**0** b16=**0** b17=**0** b18=**0** b19=**0**

a0=**0** a1=**0** a2=**0** a3=**0** ac1=**0** ac2=**0** a6=**0** a7=**0** a8=**0** a9=**0** a10=**0** a11=**0** a12=**0** a13=**0** a14=**0** a15=**0** a16=**0** a17=**0** a18=**0** a19=**0** alpha = **1**;

xc1=**0**; xc2=**0**;

if (city eq **1**) then xc1=**1**;

if (city eq **2**) then xc2=**1**;

/* linear predictor for the inflation probability */

linpinfl = a0 + a1*sex + a2*order + a3*mar + ac1*xc1 + ac2*xc2 + a6*con + a7*edu + a8*average + a9*interest + a10*optimistic + a11*boygirl + a12*breakdown + a13*hetero + a14*homo + a15*drug + a16*suicidetlt + a17*suicidealt + a18*social + a19*GHQ28gr;

infprob = **1**/(**1**+exp(-linpinfl));

/* negative binomial with mean-dispersion */

lambda = exp(b0 + b1*sex + b2*order + b3*mar + bc1*xc1 + bc2*xc2 + b6*con + b7*edu + b8*average + b9*interest + b10*optimistic + b11*boygirl + b12*breakdown + b13*hetero + b14*homo + b15*drug + b16*suicidetlt + b17*suicidealt + b18*social + b19*GHQ28gr);

/* Build the ZINB log likelihood */

m = **1**/alpha;

p = **1**/(**1**+alpha*lambda);

if number=**0** then

ll = log(infprob + (**1**-infprob)*(p**m));

else ll = log(**1**-infprob) + log(gamma(m + number)) - log(gamma(number + **1**))

- log(gamma(m)) + m*log(p) + number*log(**1**-p);

model number ~ general(ll);

**run**;

**Code_** **ZIP**

**proc** **nlmixed** data=data;

parameters b0=**0** b1=**0** b2=**0** b3=**0** bc1=**0** bc2=**0** b6=**0** b7=**0** b8=**0** b9=**0** b10=**0** b11=**0** b12=**0** b13=**0** b14=**0** b15=**0** b16=**0** b17=**0** b18=**0** b19=**0**

a0=**0** a1=**0** a2=**0** a3=**0** ac1=**0** ac2=**0** a6=**0** a7=**0** a8=**0** a9=**0** a10=**0** a11=**0** a12=**0** a13=**0** a14=**0** a15=**0** a16=**0** a17=**0** a18=**0** a19=**0**;

xc1=**0**; xc2=**0**;

if (city eq **1**) then xc1=**1**;

if (city eq **2**) then xc2=**1**;

linpinfl = a0 + a1*sex + a2*order + a3*mar + ac1*xc1 + ac2*xc2 + a6*con + a7*edu + a8*average + a9*interest + a10*optimistic + a11*boygirl + a12*breakdown + a13*hetero + a14*homo + a15*drug + a16*suicidetlt + a17*suicidealt + a18*social + a19*GHQ28gr;

infprob = **1**/(**1**+exp(-linpinfl));

/* Poisson mean */

lambda = exp(b0 + b1*sex + b2*order + b3*mar + bc1*xc1 + bc2*xc2 + b6*con + b7*edu + b8* average + b9*interest + b10*optimistic + b11*boygirl + b12*breakdown + b13*hetero + b14*homo + b15*drug + b16*suicidetlt + b17*suicidealt + b18*social + b19*GHQ28gr);

/* Build the ZIP log likelihood */

if number=**0** then

ll = log(infprob + (**1**-infprob)*exp(-lambda));

else ll = log((**1**-infprob)) - lambda + number *log(lambda) - lgamma(number + **1**);

model number ~ general(ll);

**run**;
